# Negative plasma Epstein-Barr virus DNA nasopharyngeal carcinoma in an endemic region and its influence on liquid biopsy screening programmes

**DOI:** 10.1038/s41416-019-0575-6

**Published:** 2019-09-17

**Authors:** John Malcolm Nicholls, Victor Ho-Fun Lee, Sik-Kwan Chan, Ka-Chun Tsang, Cheuk-Wai Choi, Dora Lai-Wan Kwong, Ka-On Lam, Sum-Yin Chan, Chi-Chung Tong, Tsz-Him So, To-Wai Leung, Mai-Yee Luk, Pek-Lan Khong, Anne Wing-Mui Lee

**Affiliations:** 1 Department of Pathology, Li Ka Shing Faculty of Medicine, The University of Hong Kong, Pok Fu Lam, Hong Kong; 2 Department of Clinical Oncology, Li Ka Shing Faculty of Medicine, The University of Hong Kong, Pok Fu Lam, Hong Kong; 3 Clinical Oncology Center, The University of Hong Kong-Shenzhen Hospital, Shenzhen, China; 4 Department of Diagnostic Radiology, Li Ka Shing Faculty of Medicine, The University of Hong Kong, Pok Fu Lam, Hong Kong

**Keywords:** Tumour biomarkers, Cancer screening, Head and neck cancer

## Abstract

**Background:**

Epstein-Barr virus (EBV)-associated nasopharyngeal carcinoma (NPC) in endemic regions may have undetectable plasma EBV DNA.

**Methods:**

We prospectively recruited 518 patients with non-metastatic NPC and measured their pre-treatment plasma EBV DNA. The stage distribution and prognosis between pre-treatment plasma EBV DNA-negative (0–20 copies/ml) and EBV DNA-positive (>20 copies/ml) patients following radical treatment were compared.

**Results:**

Seventy-eight patients (15.1%) were plasma EBV DNA-negative, and 62 in this subset (12.0%) had 0 copy/ml. Only 23/78 (29.5%) plasma EBV DNA-negative patients with advanced NPC (stage III-IVA) had strong EBV encoded RNA (EBER) positivity (score 3) in their tumours compared to 342/440 (77.7%) EBV DNA-positive patients of the same stages (*p* < 0.001). Though EBV DNA-negative patients had more early-stage disease (*p* < 0.001) and smaller volumes of the primary tumour and the positive neck nodes (*p* < 0.001), they had similar 5-year overall survival and cancer-specific survival to those EBV DNA-positive counterparts by stage. Similar results were also seen when plasma EBV DNA cut-off was set at 0 copy/ml.

**Conclusions:**

Patients with low-volume NPC may not be identified by plasma/serum tumour markers and caution should be taken in its utility as a screening tool for NPC even in endemic regions.

**Clinical trial registration:**

Clinicaltrials.gov Identifier: NCT02476669.

## Introduction

Nasopharyngeal carcinoma (NPC) is endemic in southern China including Hong Kong.^1^ Over the past 20 years, plasma Epstein-Barr Virus (EBV) deoxyribonucleic acid (DNA) has been advocated for the diagnosis of NPC. The rationale for this methodology has been based on the concept that since EBV has been associated with NPC, the circulating viral or tumour associated DNA (ctDNA) may be detected in the blood as a measure of tumour presence or disease burden. However, in NPC there is a sensitivity between 53 and 96%,^2^ and this wide variation has been attributed to three main factors—the analytical method of detection, disease stage and the World Health Organization (WHO) histology of the disease. There has been an attempt to develop standardised protocols of analysing plasma EBV DNA,^3,4^ but meta-analyses of plasma EBV DNA in the diagnosis of NPC have not reported the proportion of plasma EBV DNA-positive patients for each WHO type, and only four studies looked at the clinical stage.^5–7^ Squamous cell carcinomas have typically been separated by WHO from non-keratinising carcinoma, as previous studies suggested that the former was usually negative for EBV by in-situ hybridisation (ISH), while the other subtypes were positive. In Hong Kong, however, tumours with squamous differentiation can be EBV positive,^8^ and this has also been shown in NPCs from Malaysia.^9^

Hong Kong is one of the first regions in the world discovering the association of plasma EBV DNA with NPC, and devised the first quantitative assay for accurate and reliable measurement.^10^ Over the years, the lowest detection limit as a representation of improved sensitivity has decreased from 60 copies/ml to 20 copies/ml,^11,12^ and in a large NPC screening programme this low detection limit has been advocated as an initial screening tool.^12^ In this publication, we focused our attention on the incidence of plasma EBV DNA-negative (≤20 copies/ml) NPC patients, their clinico-pathological characteristics and survival outcomes, in an attempt to determine whether there are unique features concerning these patients which may account for the absence of EBV DNA in their plasma. Furthermore, since there have been recent publications on the value of liquid biopsy in screening programmes,^13^ we sought to determine whether the same concerns on the usefulness of these screening programmes could be applied to NPC.

## Methods

### Study population

The study cohort comprising 518 consecutively and prospectively recruited patients with previously untreated non-metastatic NPC (i.e. stage I–IVA) enrolled in another study which investigated the role of plasma EBV DNA in proposing a new staging system (ClinicalTrials.gov NCT02476669).^14^ All biopsy-confirmed patients had complete staging investigations, including positron-emission tomography with integrated contrast-enhanced computed tomography (PET-CT) scan, magnetic resonance imaging (MRI), serum haematology, biochemistry and lactate dehydrogenase, serology for EBV immunoglobulin A (IgA) for viral capsid antigen (VCA), and plasma EBV DNA to investigate the prognostic role of plasma EBV DNA taken at several time points before and after radical treatment (described further in Supplementary Material).^15^ The protocol and assay for plasma EBV DNA extraction and quantification for all patients in this study was the same as the one devised by Lo et al.^10,12^ In brief, all patient blood samples contained in EDTA tubes were immediately stored in a 4 °C  refrigerator after blood taking from patients and they were processed for subsequent EBV DNA extraction within 4 h of blood taking in the single laboratory of our institution (further details on EBV DNA quantification and validation methods were described in Supplementary Material). A total of about 400–800 μl of plasma samples were used for DNA extraction by a QIAamp Blood Kit (Qiagen, Hilden, Germany). The exact amount of plasma was determined for calculation of EBV DNA genome copies. Circulating EBV DNA concentrations were measured using a real-time quantitative polymerase chain reaction (PCR) system with ABI Prism® 7000 Sequence Detection System (Applied Biosystems, USA) that amplified a DNA segment in the *Bam*HI-W fragment region of the EBV genome. All samples were repeated twice on the same day by the same assay for accurate quantification and the results showed that the discrepancy was less than 2% for all repeated samples. The results were expressed as EBV DNA genome copies per ml with accuracy to the nearest 0.1 copies/ml.^14^ All pre-treatment investigations were performed within 14 days of the pathological diagnosis of NPC. All patients, within 14 days of these investigations, then received radical intensity-modulated radiation therapy (IMRT) with or without concurrent chemotherapy and adjunct (induction or adjuvant) chemotherapy based on the stage of the disease according to the 7th edition of American Joint Committee on Cancer (AJCC)/Union for International Cancer Control (UICC) TNM staging system. The details of treatment and follow-up surveillance were previously described.^14,15^ The TNM stage of each patient’s disease was re-staged according to 8th edition of AJCC/UICC TNM staging system for subsequent analysis in this study.^14^

As there has been no consensus on what determines the lowest limit of plasma EBV DNA detection, we selected and analysed patients who had 0–20 copies of EBV DNA per ml in the plasma as this has been regarded as the lowest limit of detection threshold in a recently reported screening study, with plasma EBV DNA ≤20 copies/ml (i.e. 0–20 copies/ml) designated as plasma EBV-negative.^12^ In this publication, plasma EBV DNA-negative NPC and EBV DNA-positive NPC were defined as those diagnosed in patients who had pre-treatment ≤20 copies/ml and > 20 copies/ml respectively.

### Histology and ISH Epstein-Barr virus-encoded RNA (EBER) analysis

All NPCs in this study were classified according to WHO criteria into keratinising squamous cell carcinoma, non-keratinising differentiated carcinoma and non-keratinising undifferentiated carcinoma. The formalin-fixed paraffin-embedded (FFPE) tumour slides were subjected to ISH using the commercially available Inform EBER ISH probe (Ventana). They were scored based on the percentage of tumour cells positively stained with EBER as follows: 0 (no tumour cells positively stained); 1 (1–10% tumour cells positively stained); 2 (11–50% tumour cells positively stained), and 3 (>50% tumour cells positively stained) blindly by 2 independent pathologist and oncologist (JN and VL), suggested by Bar-Sela et al.^16^ An example of microscopic appearance of each EBER intensity by ISH (0–3) in 4 patients of this study cohort was shown (Fig. S1). An excellent agreement (Cohen’s Kappa 0.83) was observed and any discrepancy in scoring was resolved by consensus. An additional 85 cases of histologically confirmed NPC from 2013 to 2017 with plasma EBV DNA titres ≥30 copies/ml were used as positive controls.

### Tumour volume analysis

The pre-treatment gross tumour volumes (GTV) of the primary nasopharyngeal tumour (GTV_P) and the radiologically positive neck nodes (GTV_N) of the contrast-enhanced PET-CT images of 3 mm slice thickness with reference to the co-registered MRI images were contoured manually by the treating clinical oncologists in Eclipse Treatment Planning System version 13.0 (Palo Alto, USA), which was also used for subsequent IMRT optimisation, as previously described.^14,15^ The resulting sum of the areas of the GTV_P and GTV_N was calculated by this treatment planning system to generate the respective volumes.

### Statistical analysis

The pre-specified survival endpoints in this study included progression-free survival (PFS), overall survival (OS) and cancer-specific survival (CSS) as we defined previously.^14,15^ Kaplan–Meier methods were performed for these survival outcomes. Log-rank tests were employed to compare survival differences between plasma EBV DNA-negative and EBV DNA-positive patients. Association between EBER scores and T-, N- and overall stage of NPC as well as pre-treatment plasma EBV DNA subgroups (0–20 copies/ml vs >20 copies/ml and 0 copy/ml vs >0 copy/ml) was performed by Chi-square tests. Statistical significance was defined as *p* *<* 0.05 (two-sided). All statistical analyses were performed by Statistical Package for Social Sciences (SPSS) version 24.

## Results

**Fig. 1:**
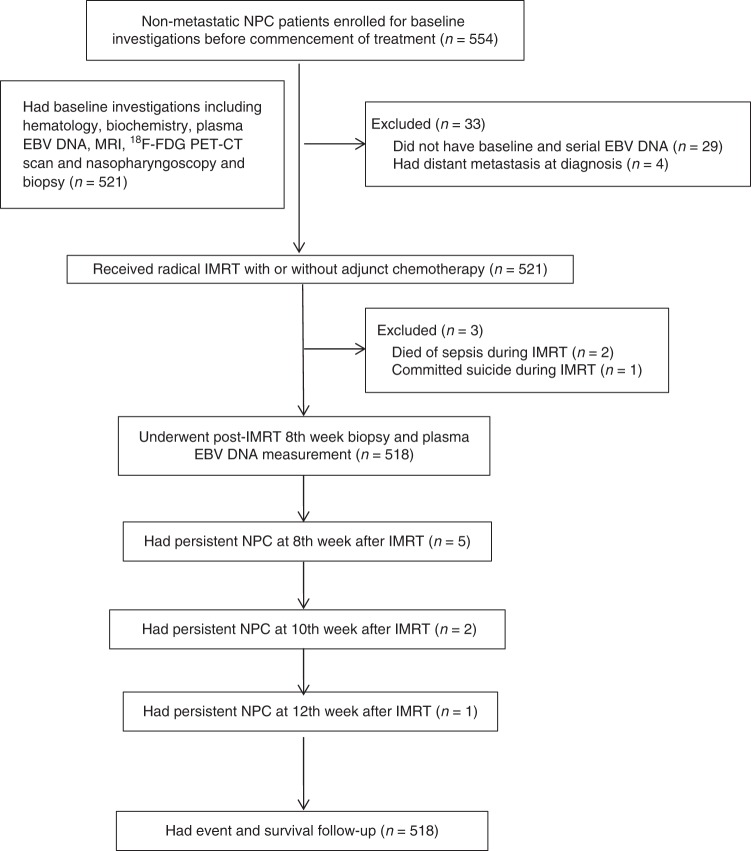
Study flowchart

From October 2010 to May 2016, 518 patients were prospectively recruited with their dispositions shown (Fig. 1, Table 1). Seventy-eight (15.1%) patients were classified as pre-treatment plasma EBV DNA-negative (i.e. ≤20 copies/ml). Sixty-two (79.5%) patients had 0 copy/ml and 16 (20.5%) had 1–20 copies/ml of plasma EBV DNA before treatment. Pre-treatment plasma EBV DNA correlated very well with GTV_P, GTV_N, GTV_T + N and serum lactate dehydrogenase (all *p* *<* 0.01) (Fig. S2). However, no correlation was identified between pre-treatment plasma EBV DNA and GTV_P (*p* *=* 0.936), GTV_N (*p* = 0.900) and GTV_T+N (*p* *=* 0.910) in plasma EBV DNA-negative patients. Plasma EBV DNA-negative patients tended to have an earlier stage of their NPC, as well as smaller volumes of the primary tumour in the nasopharynx and the positive neck nodes. The overall stage distribution of these 78 plasma EBV DNA-negative patients was: stage I in 18 (23.1%); stage II in 17 (21.8%); stage III in 34 (43.6%) and stage IVA in 9 (11.5%). No plasma EBV DNA-negative patients had stage IVB metastatic disease at the time of initial diagnosis. The T- and N-classification of the plasma EBV DNA-negative patients were shown (Table S1).

**Table 1:** Patient characteristics at baseline based on 8th edition of AJCC/UICC staging classification stratified by pre-treatment plasma EBV DNA (0–20 copies/ml vs >20 copies/ml)

Characteristic	Patients, No. (%)			*p*
	Total (*n* = 518)	Pre-treatment plasma EBV DNA		
		0–20 copies/ml (*n* = 78)	> 20 copies/ml (*n* = 440)	
Median age in years (range)	53 (16–90)	54 (16–86)	53 (16–90)	0.983
Male/female	385 (74.3)/133 (25.7)	61 (78.2)/17 (21.8)	324 (73.6)/116 (26.4)	0.395
Histology				0.405
Keratinising squamous cell carcinoma	1 (0.2)	0 (0)	1 (0.2)	
Non-keratinising differentiated carcinoma	9 (1.7)	0 (0)	9 (2.0)	
Non-keratinising undifferentiated carcinoma	508 (98.1)	78 (100)	430 (97.8)	
ECOG performance status				0.507
0	80 (15.4)	14 (17.9)	66 (15)	
1	438 (84.6)	64 (82.1)	374 (85)	
T-classification				<0.001
T1	147 (28.4)	40 (51.3)	107 (24.3)	
T2	72 (13.9)	8 (10.3)	64 (14.5)	
T3	234 (45.2)	26 (33.3)	208 (47.3)	
T4	65 (12.5)	4 (5.1)	61 (13.9)	
N-classification				<0.001
N0	60 (11.6)	25 (32.1)	35 (8.0)	
N1	127 (24.5)	21 (26.9)	106 (24.1)	
N2	201 (38.8)	27 (34.6)	174 (39.5)	
N3	130 (25.1)	5 (6.4)	125 (28.4)	
Overall stage				<0.001
I	30 (5.8)	18 (23.1)	12 (2.7)	
II	74 (14.3)	17 (21.8)	57 (13.0)	
III	234 (45.2)	34 (43.6)	200 (45.5)	
IVA	180 (34.7)	9 (11.5)	171 (38.8)	
Laterality of primary tumour				0.796
Midline	231 (44.6)	35 (44.9)	196 (44.5)	
Left	160 (30.9)	26 (33.3)	134 (30.5)	
Right	127 (24.5)	17 (21.8)	110 (25)	
Involvement of retropharyngeal node	388 (74.9)	43 (55.1)	345 (78.4)	<0.001
Median pretreatment plasma EBV DNA in copies/millilitre (range)	588.5 (0–1143750)	0 (0–19)	895 (22–1143750)	0.001
Stage I	12 (0–315)	0 (0–19)	65 (24–315)	0.004
Stage II	321 (0–8850)	0 (0–19)	547 (22–8850)	<0.001
Stage III	494 (0–175000)	0 (0–17)	705.5 (22–175000)	<0.001
Stage IVA	2012.5 (0–1143750)	0 (0–14)	2203 (38–1143750)	0.584
Median pretreatment serum lactate dehydrogenase in international units/litre (range)	196 (109–688)	180.5 (125–310)	196 (109–688)	0.011
Stage I	179.5 (121–310)	178.5 (132–310)	190.5 (121–260)	0.655
Stage II	185.5 (140–275)	174 (143–275)	188 (140–256)	0.235
Stage III	197.5 (109–521)	191 (137–306)	198 (109–521)	0.593
Stage IVA	200 (125–688)	182 (125–254)	204 (130–688)	0.174
Median Gross tumour volume of the primary tumour (GTV_P) (cm^3^) (range)	8.7 (0–136)	4.4 (0–66.9)	10.1 (0–136)	0.004
Median Gross tumour volume of the positive neck nodes (GTV_N) (cm^3^) (range)	17.1 (0–191.3)	9.45 (0.4–191.3)	18.55 (0–168.2)	<0.001
Median Gross tumour volume of the primary tumour and the positive neck nodes (GTV_P + N) (cm^3^) (range)	31.4 (0.9–229)	16.25 (2.3–199.9)	34.9 (0.9–229)	<0.001
Radical IMRT only	71 (13.7)	26 (33.3)	45 (10.2)	<0.001
Concurrent chemoradiation	91 (17.6)	14 (17.9)	77 (17.5)	0.194
Induction chemotherapy then concurrent chemoradiation	165 (31.9)	12 (15.4)	153 (34.8)	0.022
Concurrent chemoradiation then adjuvant chemotherapy	191 (36.9)	27 (34.6)	164 (37.3)	0.199

*AJCC* American Joint Committee on Cancer, *EBV DNA* Epstein-Barr virus deoxyribonucleic acid, *ECOG* Eastern Cooperative Oncology Group, *IMRT* intensity-modulated radiation therapy, *UICC* Union for International Cancer Control

**Fig. 2:**
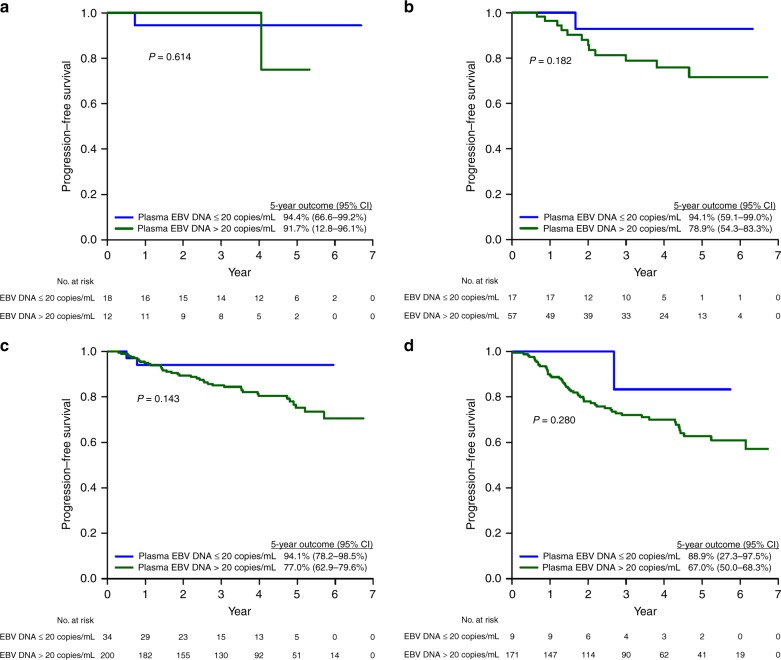
Progression-free survival of NPC patients in the study population stratified by pre-treatment plasma EBV DNA (0–20 copies/ml vs >20 copies/ml). **a** Stage I. **b** Stage II. **c** Stage III. **d** Stage IVA

**Fig. 3:**
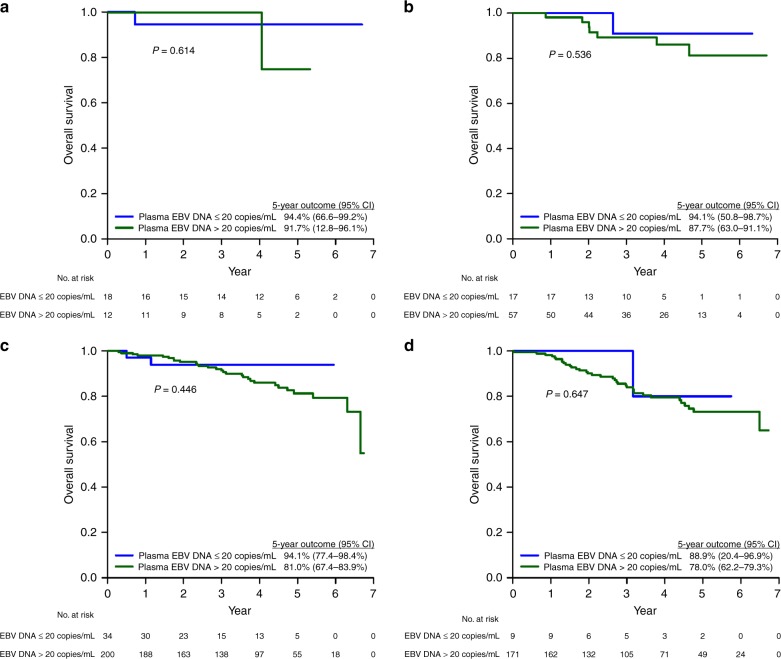
Overall survival of NPC patients in the study population stratified by pre-treatment plasma EBV DNA (0–20 copies/ml vs >20 copies/ml). **a** Stage I. **b** Stage II. **c** Stage III. **d** Stage IVA

**Fig. 4:**
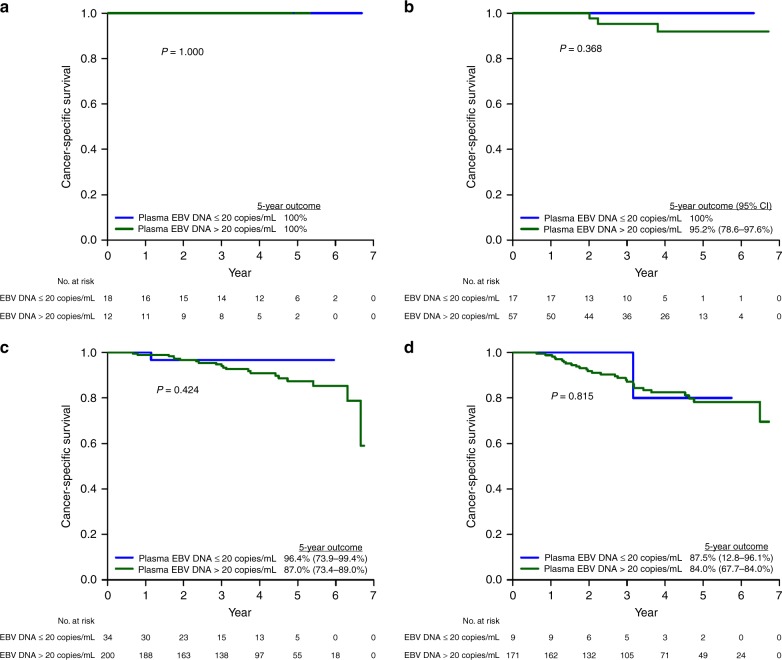
Cancer-specific survival of NPC patients in the study population stratified by pre-treatment plasma EBV DNA (0–20 copies/ml vs >20 copies/ml). **a** Stage I. **b** Stage II. **c** stage III. **d** stage IVA

After a median follow-up of 5.2 years (range 1.2–6.4 years), the 5-year PFS, OS and CSS of the whole population were 72.9%, 79.8% and 86.1%, respectively. Except for PFS (92.7% vs 70.0%, 95% CI = 83.3–97.2% vs 73.1–82.8%, *p* *=* 0.023), OS (91.9% vs 78.1%, 95% CI = 81.1–97.1% vs 73.1–82.8%, *p* *=* 0.242) and CSS (96.2% vs 84.6%, 95% CI = 85.2–99.0% vs 80.0–88.3%, *p* *=* 0.293) of the plasma EBV DNA-negative patients were not statistically better than their EBV DNA-positive counterparts (Table S2). When compared by stage, their survival outcomes (PFS, OS and CSS) were also not different from the plasma EBV DNA-positive patients (Figs. 2–4).

Since there were 62 and 16 patients who had 0 copies/ml and 1–20 copies/ml of pre-treatment plasma EBV DNA, respectively, we also compared the stage distribution, tumour and nodal volumes and survival endpoints with those who had pre-treatment plasma EBV DNA >20 copy/ml (Tables S3–S6). Again, patients with pre-treatment plasma EBV DNA 0 copy/ml had more early-stage disease, smaller tumour and nodal volumes but similar survival outcomes (except PFS) as compared to those who had pre-treatment plasma EBV DNA >0 copy/ml (Figs. S3–S5 and Table S7).

### Association of EBER with plasma EBV DNA and overall stage of disease

The association of EBER by ISH with plasma EBV DNA and NPC T-, N- and overall stage was shown (Tables S8–S13). Though statistical significances on the association between EBER scores and overall stage were identified in the whole study population and plasma EBV DNA-positive patients, they were not detected in plasma EBV DNA-negative patients. Intriguingly, only 23 out of 78 (29.5%) of advanced NPC (stage III-IVA) patients who were plasma EBV DNA-negative had diffuse strong EBER positivity (score 3) in their tumours, as compared to 342 of 440 (77.7%) plasma EBV DNA-positive patients of the same stages (*p* *<* 0.001). Plasma EBV DNA-negative patients were also associated with a lower EBER intensity in their tumours (*p* < 0.001) (Table S11). The results were also similar when 0 copy/ml was set as the cut-off: 41 of 62 (66.1%) had EBER score 3 in patients with plasma EBV DNA 0 copy/ml compared to 412 of 456 (90.4%) in patients with plasma EBV DNA > 0 copy/ml (*p* *<* 0.001) (Tables S11–S13).

## Discussion

In view of the close association between NPC and EBV, antibodies to EBV have been used for NPC screening and treatment monitoring, and to differentiate NPCs from other head and neck cancers.^17,18^ Immunohistochemistry and ISH studies conclusively demonstrated that the EBV genome was incorporated into the tumour cells,^19–21^ resulting in EBV being classified as an oncogenic virus.^22^

In the late 1990’s several studies showed that cancer-derived cells or DNA could be detected in the blood of cancer patients,^23,24^ and this was applied to NPC first by Mutirangura et al and later by Lo et al.^25,26^ Using the BamH1-W and EBNA1 targets to amplify, 55 of 57 patients had plasma EBV DNA detected compared to 3 of 43 normal individuals, giving a detection rate of 96%. The false positive rate was 8%. A recent review, however, showed that the sensitivity has ranged from 53–96%.^2^

The superiority of plasma EBV DNA over serum IgA VCA was later shown in 2004 by Shao et al, who also demonstrated that plasma EBV DNA correlated with the TNM stage,^27^ though IgA VCA was better at detecting stage I compared to stage IV disease. Plasma EBV DNA also appeared to be linearly related to T-stage which was suggested to be a measure of tumour burden. Leung et al in the same year also investigated the accuracy of combination of plasma EBV DNA and IgA VCA in detecting NPC.^11^ Using a detection limit of 60 copies/ml, EBV DNA was detected in 132/139 patients, with an increased sensitivity seen in later stages (87/89) than early stages (45/50). The wide range of sensitivity previously reported may be due to many factors such as extraction technique, amplification technique and whether the BamH1-W region (which has more repeats for detection, but random in the number of repeats), or single-copy genes (e.g. EBNA1) are used as targets.^28^ Other potential factors that may contribute to assay include failed extraction, inhibition, patient age and the ambient temperature when blood is taken from patients.^29,30^ A previous report has shown low copy number of EBV in 3–7% of the general population when the BamH1-W target was used, and 2.03% when the EBNA1 target was used (1–512 copies/ml), so at these low numbers there is an overlap between normal subjects and patients with stage I NPC.^31^

In the screening programme performed by Chan et al., 9% of normal individuals had detectable plasma EBV DNA in the winter season and 5% in the summer, in which 20 copies/ml was the lowest limit of detection.^30^ On the other hand, when 0 copies/ml was used as a cut-off, plasma EBV DNA identified 17/24 NPC patients in a non-endemic (but high-risk population) area.^32^

The challenge of a screening tool for cancer is the balance between sensitivity and specificity. Recent publications have addressed the community acceptance of a false positive rate in cancer screening programs,^33,34^ where it has been recognised that there is a negative psychological outcome in patients with false positive screening in breast and lung cancer.^35,36^ Yip and colleagues commented that the lowest sensitivity of plasma EBV DNA for NPC is in the stage I patients—the major targets of screening programmes, but this review has been hampered by the very small number of patients included with this stage (ranging from 1 to 14 patients in each respective study) and the wide range of median EBV DNA levels present in these patients (14–2500 copies/ml).^37^ In a recent review of the usefulness of liquid biopsy for the detection of tumours,^13^ it was suggested that one of the factors affecting the effectiveness of a cancer screening test was the weight, or volume of the tumour. In patients with a tumour weight of 10 g and sampling 10 ml of blood, the cancer screening test appears effective, but in patients with tumour weight less than that, the effectiveness was called into question. Since Table 1 shows that the primary tumour GTV of our EBV negative cohort was 4.4 g compared to 10 g for the EBV positive patients, it is not surprising that the no EBV can be detected in the plasma. Further data has demonstrated that when the fraction of tumour DNA drops below 0.01%, then the use of 10 ml of blood (4 ml plasma) will not contain a single cancer genome.^13,38^

Since detection of plasma EBV may be problematic in patients with low volume early-stage disease, one would expect a better correlation in high tumour volume patients and Figure S2 indeed does show a good correlation. Our ISH results show that one possible hypothesis to explain this low or absent detectable plasma EBV DNA is because of tumour heterogeneity in EBV copy number. The studies from Chan and Lo et al. have assumed that each tumour cell contains 50 copies EBV/cell.^12^ However, the original publication, found a great range of EBV genome equivalents (2–137) in NPC patients.^39^ In addition to variable but multiple copies of the genome in each cell, there are also variable but multiple copies of the promoter Wp within each genome.^40,41^ The standard B95.8 cell line contains 11 reiterations of Bam-W, Raji cell line 7 copies and clinical isolates a mean of 6, with a range of 5–11.^42^ We found in many tumours that had no detectable plasma EBV DNA there was a degree of variable EBER signal in the tumour cells—in some cases the signal was located within the nucleolus, in others the whole nucleus seemed to have positive signal, and some cells had no signal at all.

Our study demonstrated that 15.1% of histologically confirmed NPC patients were plasma EBV DNA-negative and that in these plasma EBV DNA-negative patients, 99.8% were either non-keratinising differentiated carcinoma or undifferentiated carcinoma. In response to a review article indicating 17.2%–29.3% of NPC patients from endemic countries had undetectable plasma EBV at initial diagnosis,^43,44^ Le and colleagues suggested that some NPC may have a non-EBV origin, and the method of analysis may not be consistent.^45^ With respect to the possibility that NPCs may have a non-EBV origin, we found that almost all NPCs had a positive EBER ISH signal, but the signal intensity was only associated with plasma EBV DNA in those patients who had plasma EBV DNA >20 copies/ml. Inconsistent sample analysis is not likely as the samples in the current study were analysed by the same methodology used by Chan et al. in their screening programme.^12^ Technical error is also unlikely as all our cases were handled by the same laboratory with the same EBV target. All samples were analysed within 1 day of collection, so sample deterioration is unlikely to be a major factor in the evaluation of plasma EBV DNA.^46^ All patients who had negative plasma EBV DNA at the time of diagnosis also had negative plasma EBV DNA at subsequent testing and follow up. Similarly, all patients who had pre-treatment detectable plasma EBV DNA had its significant drop following treatment. It should be noted that even in an endemic region when the method of Lo et al. was used, 3/40 (7.5%) cases of NPC had 0 copy/ml, and one of these negative cases had a positive detection when the same sample was analysed by two other laboratories.^4^

We demonstrated that EBER was poorly expressed in patients with low pre-treatment plasma EBV DNA (regardless of the cut-off set at 0 or 20 copies/ml). It was also poorly associated with T-, N- and overall stage of their disease even when it is advanced stage. In addition, EBER expression levels were strongly associated with pre-treatment plasma EBV DNA. Yet about half of these patients presented with advanced stage III to IVA disease at diagnosis. It can be inferred that EBV genome may be poorly incorporated in the tumour cells in this subgroup of patients leading to impaired expression of the EBER and plasma EBV DNA production. Since EBER has been demonstrated to influence the expression of RIG-I, inflammatory mediators and thus tumour progression,^47^ the link between EBER and tumour stage may be a reflection of the EBER induced stromal response to tumour growth leading to cell death and viral DNA release. The use of only a few slides from each patient’s FFPE tumour samples for subsequent ISH for EBER which may not give an overall picture of the extent of EBV genome incorporation into every tumour cell is one study limitation, though we have already selected the most representative slides containing the greatest number of tumour cells to ensure consistent EBER scoring.

Though plasma EBV DNA has been proposed as a population screening tool for NPC by Chan et al.,^12^ 62/518 (11%) of patients had 0 copy, and 78/518 (15%) of patients had between 0 and 20 copies/ml in our study. It appears that if this was used solely for screening in the general population, it would miss more than 130 patients with NPC each year in Hong Kong with a 7.5 million population.^48^ In other words, we can miss 60.0%, 23.0%, 14.5% and 5.0% of stage I, II, III and IVA NPC if only plasma EBV DNA was used as the population screening tool, running into a risk of delayed diagnosis and treatment, and the survival of these plasma EBV DNA-negative patients by stage was not better than those who were EBV DNA-positive, as shown in our study.

## Conclusions

In conclusion, it was not uncommon for previously untreated NPC patients in endemic regions to have plasma EBV DNA below or close to the lowest limit of detection,^49,50^ and while plasma EBV DNA has been shown to be a reliable predictive and prognostic factor, our findings reinforced the concerns of previous authors in adopting its use for the primary diagnosis or screening of NPC in a general population.^25,26^ The use of EBV as a “liquid biopsy” tool thus has many of the problems associated with other cancer screening tools in terms of both sensitivity and specificity for early cancer detection and additional investigations are warranted if NPC has to be safely ruled out.

## Supplementary information

Supplementary Material

## Data Availability

The datasets used and/or analysed during the current study are available from the corresponding author on reasonable request.

## References

[1] Lee AW, Ma BB, Ng WT, Chan AT (2015). Management of nasopharyngeal carcinoma: current practice and future perspective. J. Clin. Oncol..

[2] Fung SY, Lam JW, Chan KC (2016). Clinical utility of circulating Epstein-Barr virus DNA analysis for the management of nasopharyngeal carcinoma. Chin. Clin. Oncol..

[3] Kim K. Y., Le Q. T., Yom S. S., Pinsky B. A., Bratman S. V., Ng R. H. et al. Current state of PCR-based Epstein-Barr virus DNA testing for nasopharyngeal cancer. *J. Natl Cancer Inst.* **109**, djx007 (2017). 10.1093/jnci/djx007 PMC6279258 28376165

[4] Le QT, Zhang Q, Cao H, Cheng AJ, Pinsky BA, Hong RL (2013). An international collaboration to harmonize the quantitative plasma Epstein-Barr virus DNA assay for future biomarker-guided trials in nasopharyngeal carcinoma. Clin. Cancer Res..

[5] Zhang W, Chen Y, Chen L, Guo R, Zhou G, Tang L (2015). The clinical utility of plasma Epstein-Barr virus DNA assays in nasopharyngeal carcinoma: the dawn of a new era?: a systematic review and meta-analysis of 7836 cases. Med. (Baltim.).

[6] Zhang J, Shu C, Song Y, Li Q, Huang J, Ma X (2016). Epstein-Barr virus DNA level as a novel prognostic factor in nasopharyngeal carcinoma: a meta-analysis. Med. (Baltim.).

[7] Liu TB, Zheng ZH, Pan J, Pan LL, Chen LH (2017). Prognostic role of plasma Epstein-Barr virus DNA load for nasopharyngeal carcinoma: a meta-analysis. Clin. Invest Med..

[8] Nicholls JM, Agathanggelou A, Fung K, Zeng X, Niedobitek G (1997). The association of squamous cell carcinomas of the nasopharynx with Epstein-Barr virus shows geographical variation reminiscent of Burkitt’s lymphoma. J. Pathol..

[9] Pathmanathan R, Prasad U, Chandrika G, Sadler R, Flynn K, Raab-Traub N (1995). Undifferentiated, nonkeratinizing, and squamous cell carcinoma of the nasopharynx. Variants of Epstein-Barr virus-infected neoplasia. Am. J. Pathol..

[10] Lo YM, Chan LY, Chan AT, Leung SF, Lo KW, Zhang J (1999). Quantitative and temporal correlation between circulating cell-free Epstein-Barr virus DNA and tumor recurrence in nasopharyngeal carcinoma. Cancer Res..

[11] Leung SF, Tam JS, Chan TC, Zee B, Chan LY, Huang DP (2004). Improved accuracy of detection of nasopharyngeal carcinoma by combined application of circulating Epstein-Barr virus DNA and anti-Epstein-Barr viral capsid antigen IgA antibody. Clin. Chem..

[12] Chan KCA, Woo JKS, King A, Zee BCY, Lam WKJ, Chan SL (2017). Analysis of plasma Epstein-Barr virus DNA to screen for nasopharyngeal cancer. N. Engl. J. Med.

[13] Fiala C, Diamandis EP (2017). Circulating tumor DNA for personalized lung cancer monitoring. BMC Med.

[14] Lee VH, Kwong DL, Leung TW, Choi CW, O’Sullivan B, Lam KO (2019). The addition of pretreatment plasma Epstein-Barr virus DNA into the8th edition of nasopharyngeal cancer TNM stage classification. Int J. Cancer.

[15] Lee VH, Kwong DL, Leung TW, Choi CW, Lai V, Ng L (2017). Prognostication of serial post-intensity-modulated radiation therapy undetectable plasma EBV DNA for nasopharyngeal carcinoma. Oncotarget.

[16] Bar-Sela G, Kuten A, Minkov I, Gov-Ari E, Ben-Izhak O (2004). Prevalence and relevance of EBV latency in nasopharyngeal carcinoma in Israel. J. Clin. Pathol..

[17] Henle G, Henle W (1976). Epstein-Barr virus-specific IgA serum antibodies as an outstanding feature of nasopharyngeal carcinoma. Int J. Cancer.

[18] Ringborg U, Henle W, Henle G, Ingimarsson S, Klein G, Silfversward C (1983). Epstein-Barr virus-specific serodiagnostic tests in carcinomas of the head and neck. Cancer.

[19] Niedobitek G, Young LS, Sam CK, Brooks L, Prasad U, Rickinson AB (1992). Expression of Epstein-Barr virus genes and of lymphocyte activation molecules in undifferentiated nasopharyngeal carcinomas. Am. J. Pathol..

[20] Wu TC, Mann RB, Epstein JI, MacMahon E, Lee WA, Charache P (1991). Abundant expression of EBER1 small nuclear RNA in nasopharyngeal carcinoma. A morphologically distinctive target for detection of Epstein-Barr virus in formalin-fixed paraffin-embedded carcinoma specimens. Am. J. Pathol..

[21] Yeung WM, Zong YS, Chiu CT, MacMahon E, Lee WA, Charache P (1993). Epstein-Barr virus carriage by nasopharyngeal carcinoma in situ. Int J. Cancer.

[22] Bouvard V, Baan R, Straif K, Grosse Y, Secretan B, El Ghissassi F (2009). A review of human carcinogens–Part B: biological agents. Lancet Oncol..

[23] Nawroz H, Koch W, Anker P, Stroun M, Sidransky D (1996). Microsatellite alterations in serum DNA of head and neck cancer patients. Nat. Med..

[24] Chen XQ, Stroun M, Magnenat JL, Nicod LP, Kurt AM, Lyautey J (1996). Microsatellite alterations in plasma DNA of small cell lung cancer patients. Nat. Med.

[25] Mutirangura A, Pornthanakasem W, Theamboonlers A, Sriuranpong V, Lertsanguansinchi P, Yenrudi S (1998). Epstein-Barr viral DNA in serum of patients with nasopharyngeal carcinoma. Clin. Cancer Res.

[26] Lo YM, Chan LY, Lo KW, Leung SF, Zhang J, Chan AT (1999). Quantitative analysis of cell-free Epstein-Barr virus DNA in plasma of patients with nasopharyngeal carcinoma. Cancer Res..

[27] Shao JY, Li YH, Gao HY, Wu QL, Cui NJ, Zhang L (2004). Comparison of plasma Epstein-Barr virus (EBV) DNA levels and serum EBV immunoglobulin A/virus capsid antigen antibody titers in patients with nasopharyngeal carcinoma. Cancer.

[28] De Paoli P, Pratesi C, Bortolin MT (2007). The Epstein Barr virus DNA levels as a tumor marker in EBV-associated cancers. J. Cancer Res Clin. Oncol..

[29] Gulley ML, Fan H, Elmore SH (2006). Validation of Roche LightCycler Epstein-Barr virus quantification reagents in a clinical laboratory setting. J. Mol. Diagn..

[30] Chan KCA, Chu SWI, Lo YMD (2018). Ambient temperature and screening for nasopharyngeal cancer. N. Engl. J. Med..

[31] Wong LP, Lai KT, Tsui E, Kwong KH, Tsang RH, Ma ES (2005). Plasma Epstein-Barr virus (EBV) DNA: role as a screening test for nasopharyngeal carcinoma (NPC)?. Int J. Cancer.

[32] O TM, Yu G, Hu K, Li JC (2007). Plasma Epstein-Barr virus immunoglobulin A and DNA for nasopharyngeal carcinoma screening in the United States. Otolaryngol. Head. Neck Surg..

[33] Van den Bruel A, Jones C, Yang Y, Oke J, Hewitson P (2015). People’s willingness to accept overdetection in cancer screening: population survey. BMJ.

[34] Rho JH, Lampe PD (2014). High-throughput analysis of plasma hybrid markers for early detection of cancers. Proteomes.

[35] Brodersen J, Siersma VD (2013). Long-term psychosocial consequences of false-positive screening mammography. Ann. Fam. Med.

[36] Wu GX, Raz DJ, Brown L, Sun V (2016). Psychological burden associated with lung cancer screening: a systematic review. Clin. Lung Cancer.

[37] Yip TT, Ngan RK, Fong AH, Law SC (2014). Application of circulating plasma/serum EBV DNA in the clinical management of nasopharyngeal carcinoma. Oral. Oncol..

[38] Abbosh C, Birkbak NJ, Wilson GA, Jamal-Hanjani M, Constantin T, Salari R (2017). Phylogenetic ctDNA analysis depicts early-stage lung cancer evolution. Nature.

[39] Andersson-Anvret M, Forsby N, Klein G, Henle W (1977). Relationship between the Epstein-Barr virus and undifferentiated nasopharyngeal carcinoma: correlated nucleic acid hybridization and histopathological examination. Int J. Cancer.

[40] Baer R, Bankier AT, Biggin MD, Deininger PL, Farrell PJ, Gibson TJ (1984). DNA sequence and expression of the B95-8 Epstein-Barr virus genome. Nature.

[41] Allan GJ, Rowe DT (1989). Size and stability of the Epstein-Barr virus major internal repeat (IR-1) in Burkitt’s lymphoma and lymphoblastoid cell lines. Virology.

[42] Sanosyan A, Fayd’herbe de Maudave A, Bollore K, Zimmermann V, Foulongne V, Van de Perre P (2017). The impact of targeting repetitive BamHI-W sequences on the sensitivity and precision of EBV DNA quantification. PLoS One.

[43] Zoto Mustafayev T, Ozyar E (2017). In Regard to Kim et al. Int J. Radiat. Oncol. Biol. Phys..

[44] Kim KY, Le QT, Yom SS, Ng RHW, Chan KCA, Bratman SV (2017). Clinical utility of Epstein-Barr virus DNA testing in the treatment of nasopharyngeal carcinoma patients. Int J. Radiat. Oncol. Biol. Phys..

[45] Le QT, Yom SS, Ng RHW, Bratman SV, Welch JJ, Chan KCA (2017). In Reply to Zoto Mustafayev and Ozyar. Int J. Radiat. Oncol. Biol. Phys..

[46] Ahsanuddin AN, Standish MC, Caliendo AM, Hill CE, Nolte FS (2008). Validation of an Epstein-Barr viral load assay using the QIAGEN Artus EBV TM PCR analyte-specific reagent. Am. J. Clin. Pathol..

[47] Duan Y, Li Z, Cheng S, Chen Y, Zhang L, He J (2015). Nasopharyngeal carcinoma progression is mediated by EBER-triggered inflammation via the RIG-I pathway. Cancer Lett..

[48] Hong Kong Cancer Registry. Available at http://www3.ha.org.hk/cancereg/default.asp. Assessed on 14 July 2018.

[49] Stevens SJ, Verkuijlen SA, Hariwiyanto B, Harijadi, Paramita DK, Fachiroh J (2006). Noninvasive diagnosis of nasopharyngeal carcinoma: nasopharyngeal brushings reveal high Epstein-Barr virus DNA load and carcinoma-specific viral BARF1 mRNA. Int J. Cancer.

[50] Tong JH, Tsang RK, Lo KW, Woo JK, Kwong J, Chan MW (2002). Quantitative Epstein-Barr virus DNA analysis and detection of gene promoter hypermethylation in nasopharyngeal (NP) brushing samples from patients with NP carcinoma. Clin. Cancer Res..

