# Supplementary material for: Negative plasma Epstein-Barr virus DNA nasopharyngeal carcinoma in an endemic region and its influence on liquid biopsy screening programmes
Source: Br J Cancer. 2019 Sep 17;121(8):690–8. doi: 10.1038/s41416-019-0575-6 (PMC6888810; doi:10.1038/s41416-019-0575-6)
Supplement: Supplementary file 1 — Supplementary Material [file 41416_2019_575_MOESM1_ESM.docx]

**Supplementary Material**

**Negative plasma Epstein-Barr virus DNA nasopharyngeal carcinoma in an endemic region and its influence on liquid biopsy screening programmes**

John Malcolm Nicholls^1^, Victor Ho-Fun Lee^2,3^, Sik-Kwan Chan^2^, Ka-Chun Tsang^2^, Cheuk-Wai Choi^2^, Dora Lai-Wan Kwong^2,3^, Ka-On Lam^2,3^, Sum-Yin Chan^2^, Chi-Chung Tong^2^, Tsz-Him So^2^, To-Wai Leung^2,3^, Mai-Yee Luk^2^, Pek-Lan Khong^4^, Anne Wing-Mui Lee^2,3^

^1^Department of Pathology, Li Ka Shing Faculty of Medicine, The University of Hong Kong, Hong Kong
^2^Department of Clinical Oncology, Li Ka Shing Faculty of Medicine, The University of Hong Kong, Hong Kong
^3^Clinical Oncology Center, The University of Hong Kong-Shenzhen Hospital, Shenzhen, China
^4^Department of Diagnostic Radiology, Li Ka Shing Faculty of Medicine, The University of Hong Kong, Hong Kong

Correspondence to: Dr. Victor Ho-Fun Lee, Department of Clinical Oncology, 1/F, Professorial Block, Queen Mary Hospital, 102 Pokfulam Road, Hong Kong.

Tel. +852 2255-4352

E-mail: [vhflee@hku.hk](mailto:vhflee@hku.hk)

**Additional methods**

**Pre-treatment investigations and details of treatment and survival follow-up**

**1. Determination of pre-treatment plasma EBV DNA titres**

In brief, four millilitres (ml) of peripheral blood was drawn and placed in an EDTA tube. All samples were immediately stored in a 4 degree Celsius refrigerator after blood taking from all patients and they were processed for subsequent EBV DNA extraction within 4 hours of blood taking from patients in the single laboratory of our institution. A total of about 400 to 800 microlitres of plasma samples were used for DNA extraction by a QIAamp Blood Kit (Qiagen, Hilden, Germany). The exact amount of plasma was determined for calculation of EBV DNA genome copies. Circulating EBV DNA concentrations were measured using a real-time quantitative polymerase chain reaction (PCR) system with ABI Prism® 7000 Sequence Detection System (Applied Biosystems, USA) that amplified a DNA segment in the *Bam*HI-W fragment region of the EBV genome. All plasma DNA samples were also subject to real-time PCR analysis for the β*-globin* gene, which gave a positive signal on all tested samples. Multiple controls without templates were also included in each analysis as negative controls. All samples were repeated twice on the same day by the same assay for accurate quantification and the results showed that the discrepancy was less than 2% for all repeated samples. All results were expressed as EBV DNA genome copies per ml with accuracy to the nearest 0.1 copies/ml. Undetectable plasma EBV DNA meant 0 copies/ml and they were used interchangeably in the main text and the Supplementary Material.

**2. IMRT planning protocol**

Every patient was immobilised in the supine position during 18F-FDG PET-CT acquisition and actual treatment by using a thermoplastic head and neck cast. A customised mouthguard was fabricated for better immobilisation. MRI images mentioned above were co-registered with the planning PET-CT images for dedicated delineation of the target volumes and organs-at-risk (OARs). OARs, including brainstem, spinal cord, globes, optic nerves, optic chiasm, lenses, temporomandibular joints, temporal lobes, auditory nerves, cochleae, mandible, oral cavity, larynx, parotid glands and vestibules were first contoured. Then gross tumour volumes (GTV) of both the primary tumour and the radiologically involved cervical nodes were outlined. Subsequently, the clinical target volume (CTV-70) for the microscopic disease spread and planning target volume containing CTV-70 with a 5-mm margin (PTV-70) to take into account physiological body motion and set-up errors were generated respectively. Another CTV-66 encompassing the high-risk areas including the posterior half of the maxillary sinuses, nasal cavities, parapharyngeal spaces, styloid processes, basiocciput, basisphenoid, clivus, foramina rotunda and ovale, pterygopalatine fossae, pterygomaxillary fissures, infraorbital fissures, cavernous sinuses, and level Ib and V nodal stations were also outlined subsequently. A corresponding PTV-66 with a 3-mm margin encompassing the CTV-66 was created by Boolean operations of the treatment planning system (Eclipse version 8.0 to 10.0 software, Eclipse Treatment Planning System, Palo Alto, CA, USA), which was also used for IMRT planning using Analytical Anisotropic Algorithm. All the targets and OARs delineation were approved by senior radiation oncologists (Victor Ho-Fun Lee, Dora Lai-Wan Kwong and To-Wai Leung) before dose optimisation for IMRT. During optimisation, the maximum dose to the brainstem, optic nerves, and chiasm was limited to 54 Gy and less than 45 Gy to the spinal cord. Allowance was given for some locally advanced tumours in which the maximum dose to the brainstem, optic nerves and chiasm could be up to 60 Gy. Efforts were also made to limit the mean dose to the parotid glands to 26 Gy and the dose to the lenses and temporal lobes to as low as could reasonably be achieved without compromising dose coverage to the PTVs. A dose of 70 Gy was prescribed to the PTV-70 and 66 Gy to the PTV-66 in 33 fractions delivered by the simultaneous accelerated radiation therapy technique (SMART). If there was no clinical nodal disease in the patients’ lower neck, either an extended IMRT field or a separate anterior field matched with a monoisocentric technique to the IMRT field above was employed based on the oncologist’s preference and 66Gy in 33 fractions were prescribed for the anterior neck field if present. All IMRT planning, dose optimisation and quality assurance was performed by a certified medical physicist (Sherry Ng) and all IMRT plans fulfilled acceptance criteria with at least 95% of PTVs having received the prescribed dose, the maximum dose of PTVs limited to 107% or below and the maximum dose of organs-at-risk within tolerance limits according to International Commission on Radiation Units and Measurements (ICRU) criteria. They were then approved by senior radiation oncologists (Victor Ho-Fun Lee, Dora Lai-Wan Kwong and To-Wai Leung) before IMRT commencement. Positional verification with on-board imaging was performed before and then daily before the first 3 fractions of IMRT followed by weekly afterwards during the whole course of IMRT, to track any anteroposterior and lateral body displacements.

**3. Chemotherapy regimens and schedules**

Treatment was based on 7^th^ edition of AJCC/UICC system and Eastern Cooperative Oncology Group (ECOG) performance status and medical comorbidities of each patient. In general, patients with stage I and II disease received IMRT alone while stage III to IVB received concurrent chemoradiation with either adjuvant or induction chemotherapy. Concurrent chemotherapy using intravenous infusional cisplatin (100mg/m^2^) was given on the first day of IMRT every 3 weeks for up to three cycles. Adjuvant chemotherapy with cisplatin (80mg/m^2^) on day 1 and 5-FU (1000mg/m^2^) from day 1 to 4 every four weeks for three cycles was started at four weeks following completion of IMRT. Patients who had their primary tumours close to critical OARs would receive three cycles of induction chemotherapy (cisplatin 100mg/m^2^ on day 1 and either 5-FU 1000mg/m^2^ from day 1 to 5 or gemcitabine 1000mg/m^2^ on day 1 and day 8, given every 3 weeks) before concurrent chemoradiation, in an attempt to achieve satisfactory tumour shrinkage so that a radical radiation dose could be delivered to the tumours with more sparing of these OARs from radiation. Those with bulky cervical nodal (≥ 3cm in diameter) stage II disease were also given concurrent chemoradiation only, at the discretion of the treating oncologist.

**4. Post-IMRT surveillance and follow-up**

Eight weeks following completion of IMRT, all patients underwent nasoendoscopy again with routine 6-site random nasopharyngeal biopsies at both roofs, lateral and posterior walls of the nasopharynx. If residual tumours were noted histologically, they needed another endoscopy and biopsies two weeks later as positive histological findings may undergo spontaneous remission with time, as we reported previously. Another endoscopy and biopsies would be performed again at 12^th^ week after completion of IMRT if there was still residual tumour at 10^th^ week after completion of IMRT. Local persistence, as we defined for more than 15 years in our institution, was tumour persistence in nasopharyngeal biopsies at 12 weeks after IMRT and patients would receive salvage treatment for instance intracavitary brachytherapy boost, stereotactic radiotherapy etc. If all 6-site biopsies were negative, patients were considered to have complete local remission. They also had plasma EBV DNA checked again on the same day of nasoendoscopy at eight weeks after IMRT completion. If their EBV DNA was still > 0 copies/ml, it would be repeated every four weeks thereafter until it was undetectable or until it was proven to have persistent local or regional disease, or distant metastasis. For patients with complete local remission, they would have regular follow-up every two to three months for any relapse and complications, as well as MRI scan every three to four months and PET-CT scan if clinically suspicious of relapse. Plasma EBV DNA was also taken at six months after IMRT and then if clinically indicated afterwards for those who had undetectable (i.e. 0 copies/ml) plasma EBV DNA eight weeks after IMRT.

**5. Survival endpoints**

Pre-specified survival endpoints in this study include progression-free survival (PFS), overall survival (OS) and cancer-specific survival (CSS). PFS was defined as the time from date of diagnosis of NPC to the date of any form of disease progression or death from any cause. OS was defined as the time from the date of diagnosis of NPC to the date of death from any cause. CSS were defined as the time from date of diagnosis of NPC to the date of cancer-related death.

**Figure S1.** In-situ hybridisation for Epstein-Barr virus encoded RNA in 4 cases of undifferentiated carcinoma of nasopharyngeal carcinoma. **a** No signal in the tumour cell nuclei. **b** Positive signal in 1–10% of tumour cell nuclei, **c** Positive signal in 11–50% of tumour cell nuclei. **d** Positive signal in > 50% of tumour cell nuclei. Blue to black represents positive signal and red is the counterstain. T = tumour cells; N = normal cells. Magnification × 200.

**
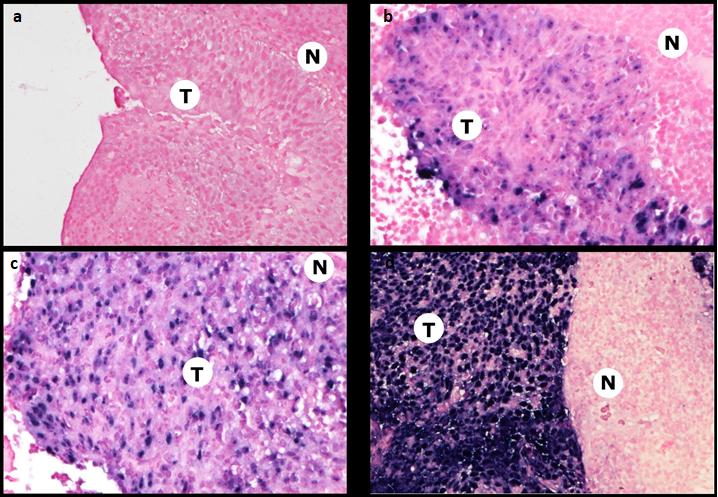
**

**Figure S2.** Correlation between pre-treatment plasma EBV DNA and gross tumour volume in the whole study population (*n* = 518). **a** Gross tumour volume of the primary tumour (GTV_P). **b** Gross tumour volume of the positive neck nodes. **c** Sum of the gross tumour volume of the primary tumour and the positive neck nodes. **d** Serum lactate dehydrogenase.

**
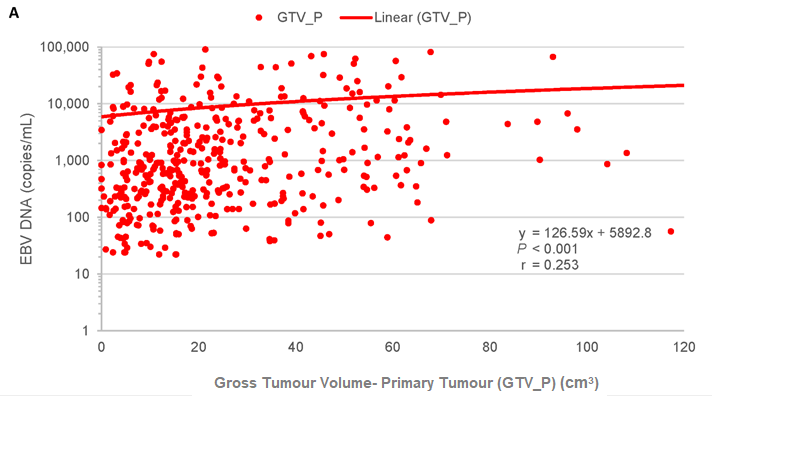
**


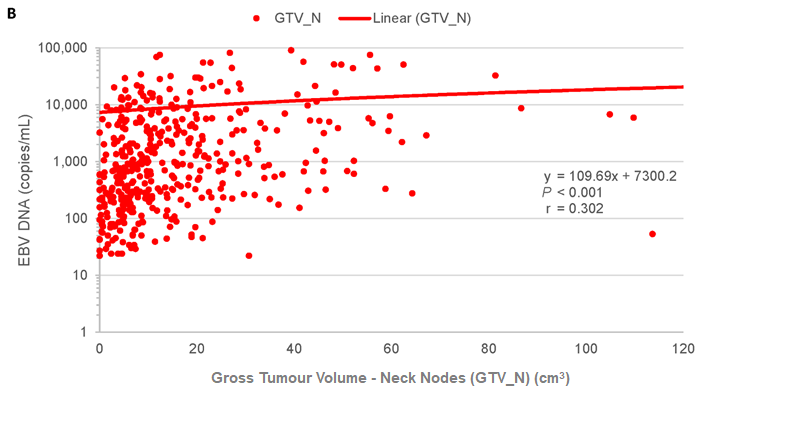


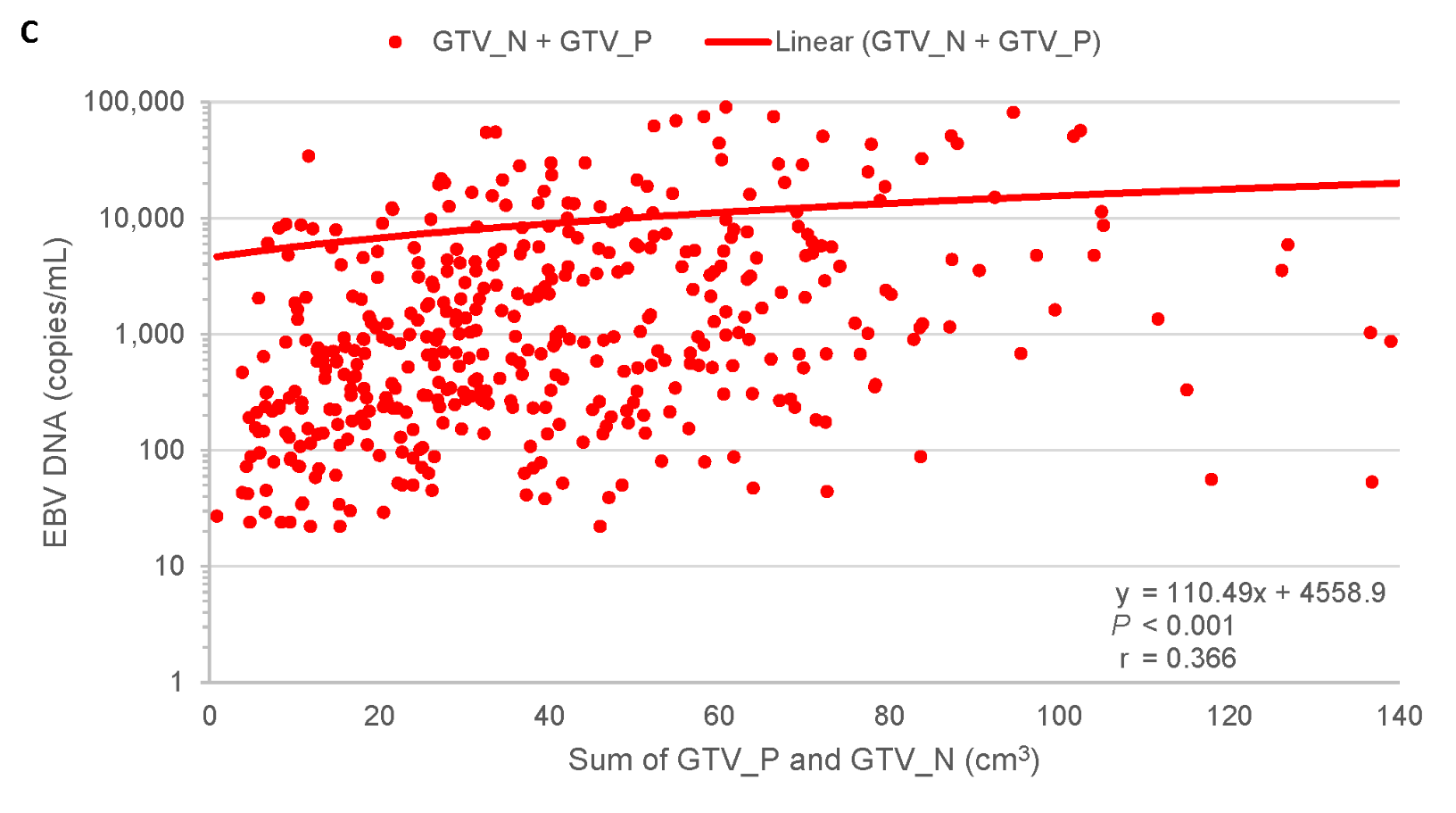


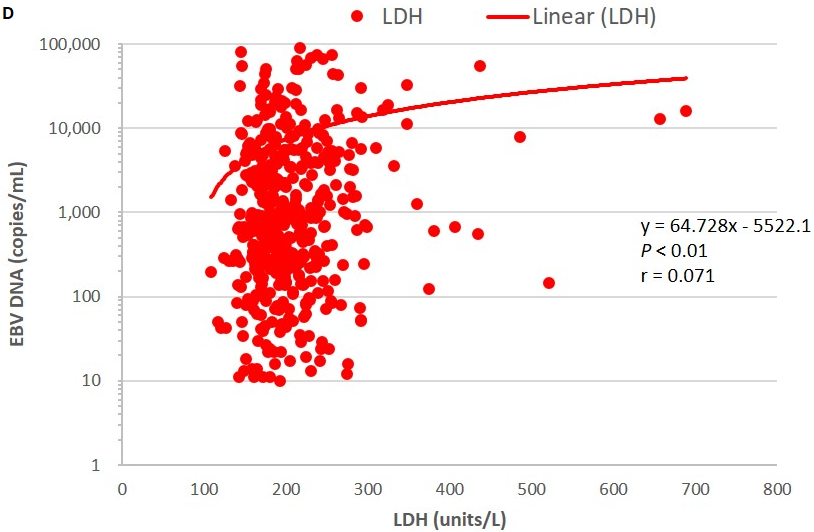


**Figure S3.** Progression-free survival of NPC patients of each stage stratified by pre-treatment plasma EBV DNA (0 copy/ml vs > 0 copy/ml). **a** Stage I. **b** Stage II. **c** Stage III. **d** Stage IVA.

**a**

**
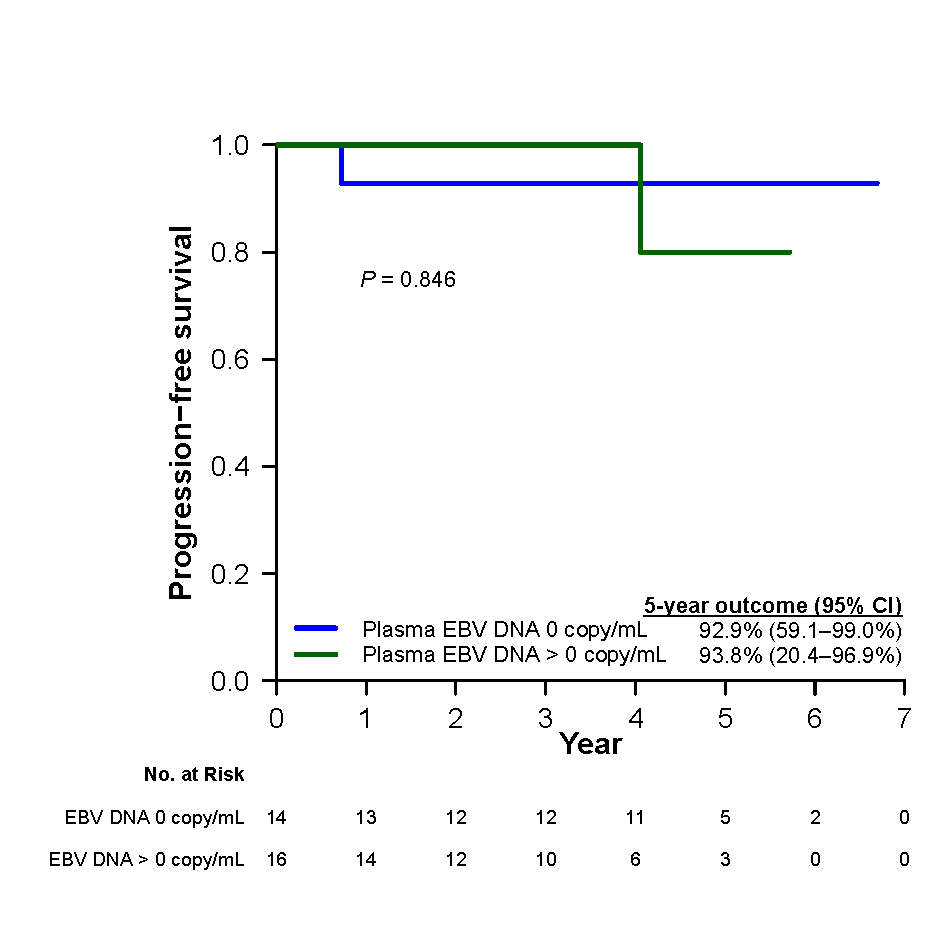
**

**b**

**
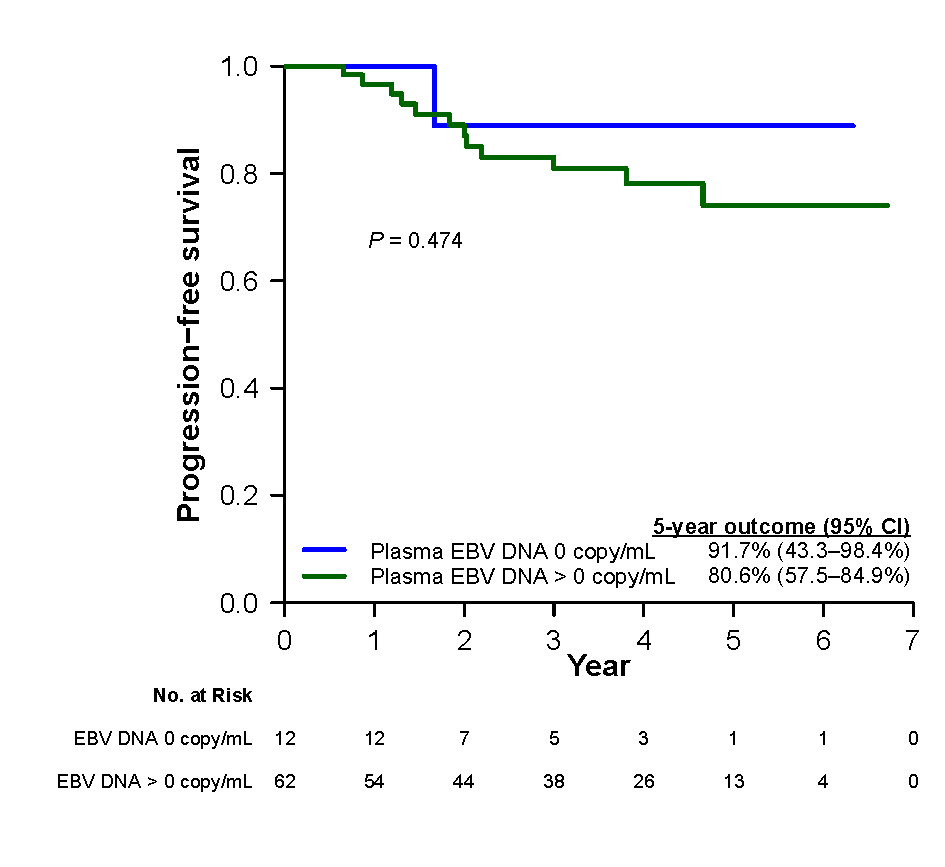
**

**c**

**
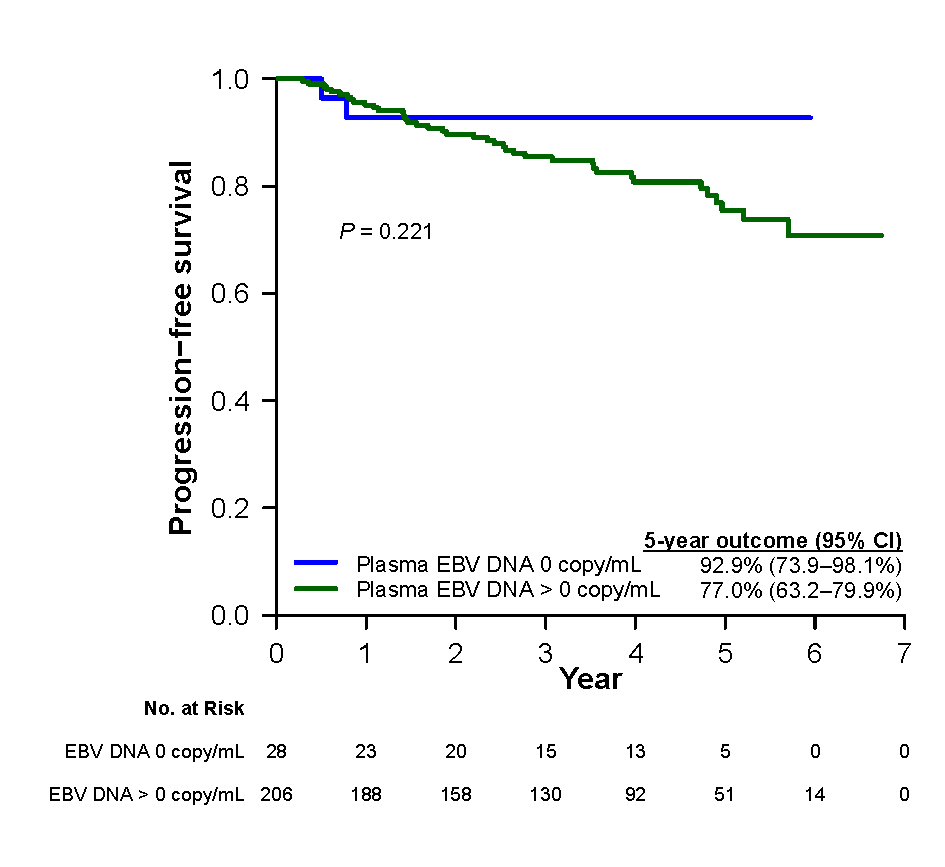
**

**d**

**
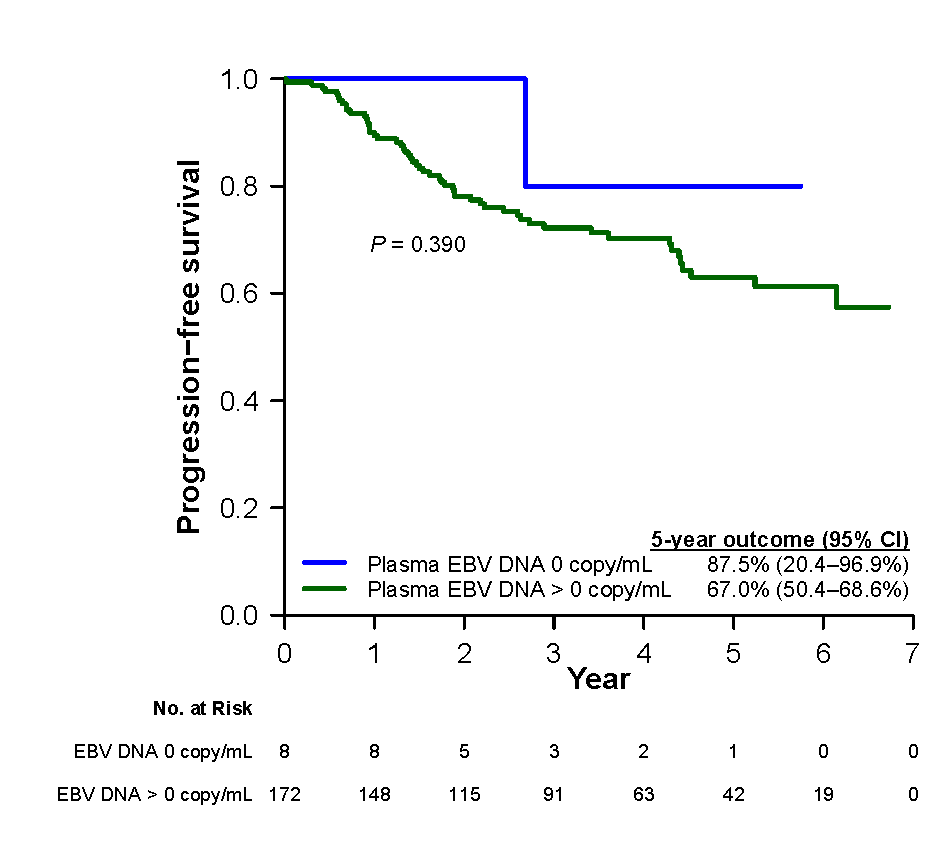
**

**Figure S4.** Overall survival of NPC patients of each stage stratified by pre-treatment plasma EBV DNA (0 copy/ml vs > 0 copy/ml). **a** Stage I. **b** Stage II. **c** Stage III. **d** Stage IVA.

**d**

**
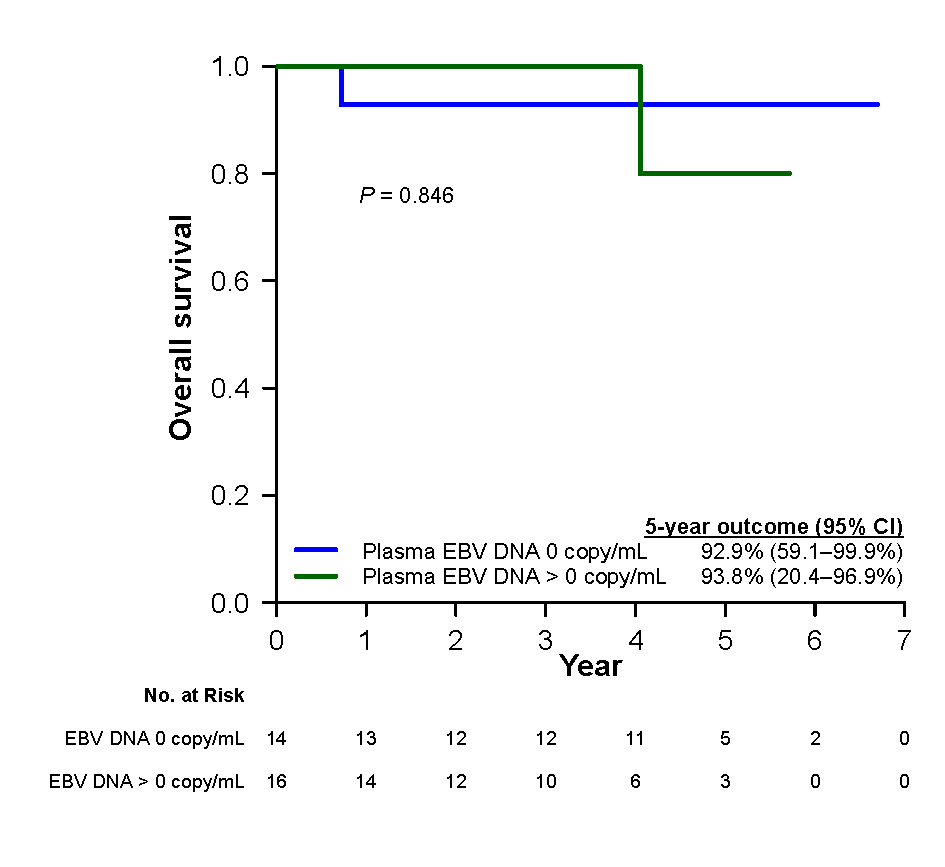
**

**b**

**
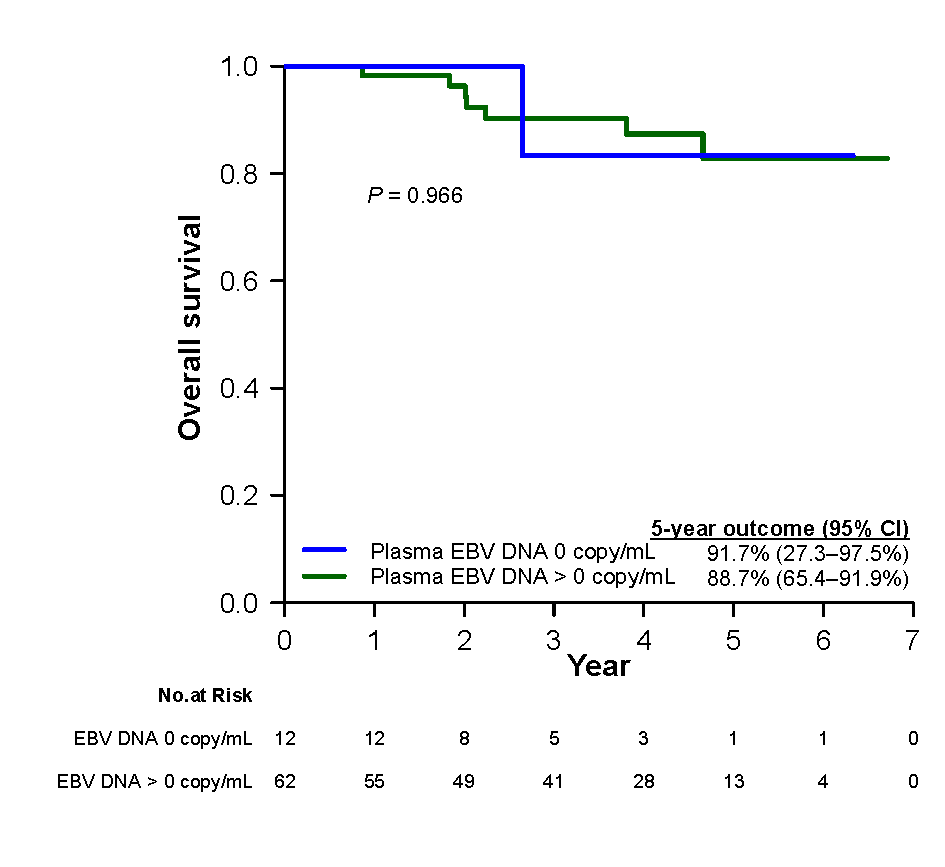
**

**c**

**
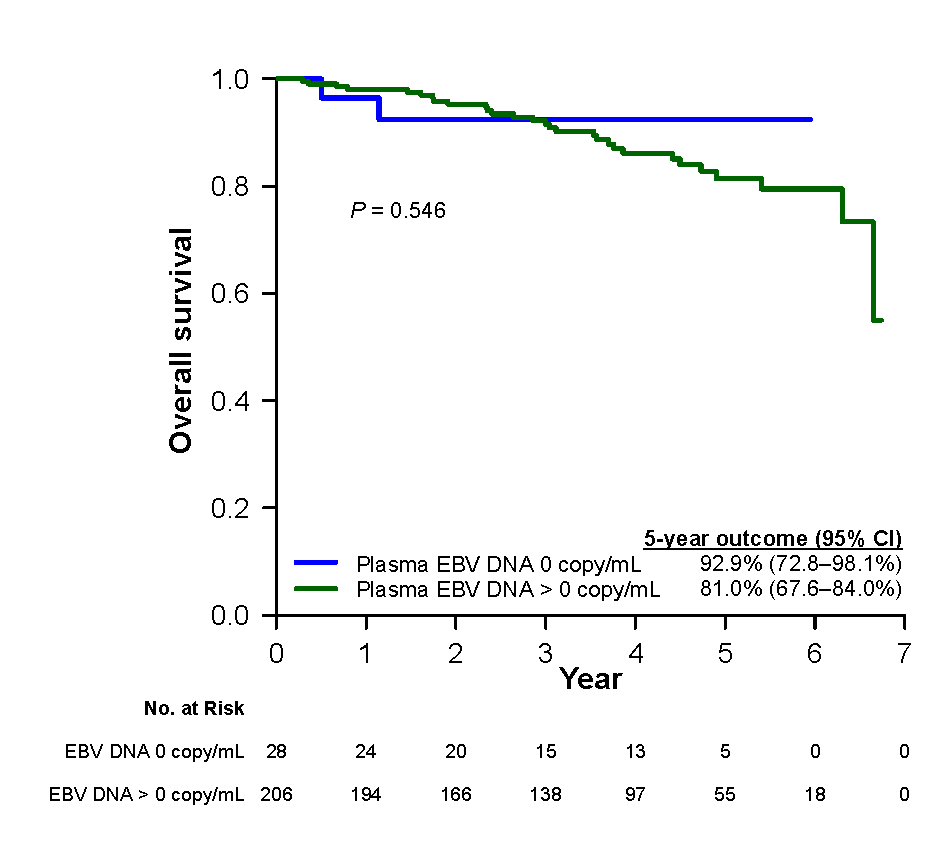
**

**d**

**
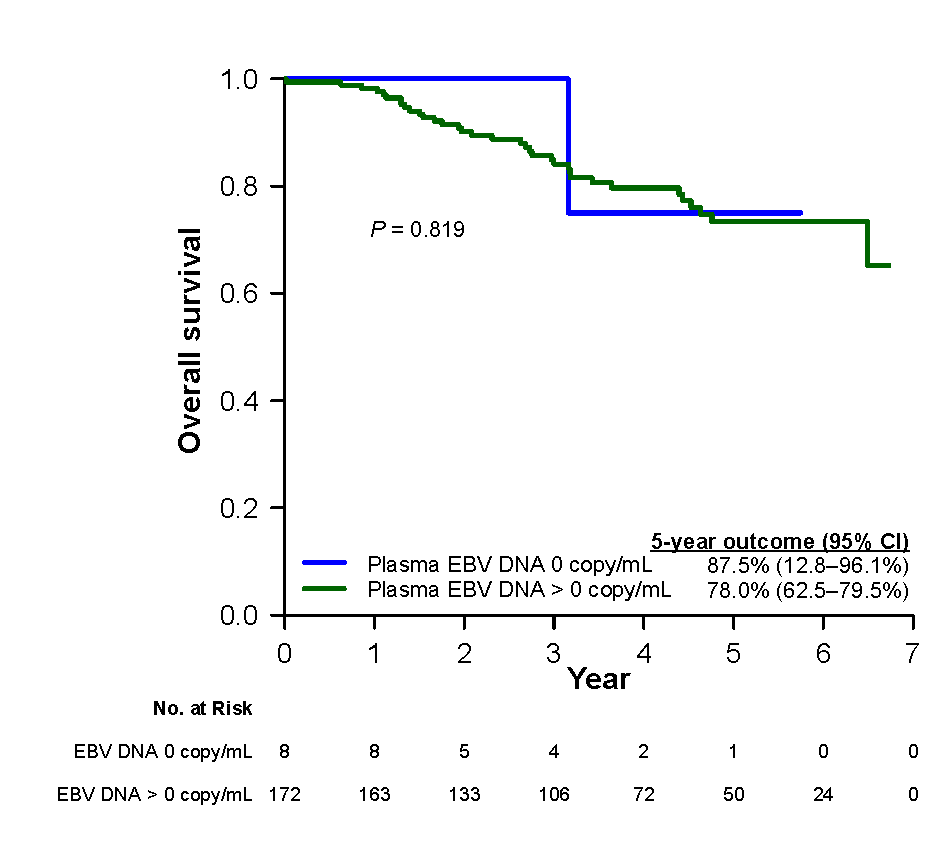
**

**Figure S5.** Cancer-specific survival of NPC patients of each stage stratified by pre-treatment plasma EBV DNA (0 copy/ml vs > 0 copy/ml). **a** Stage I. **b** Stage II. **c** Stage III. **d** Stage IVA.

**a**

**
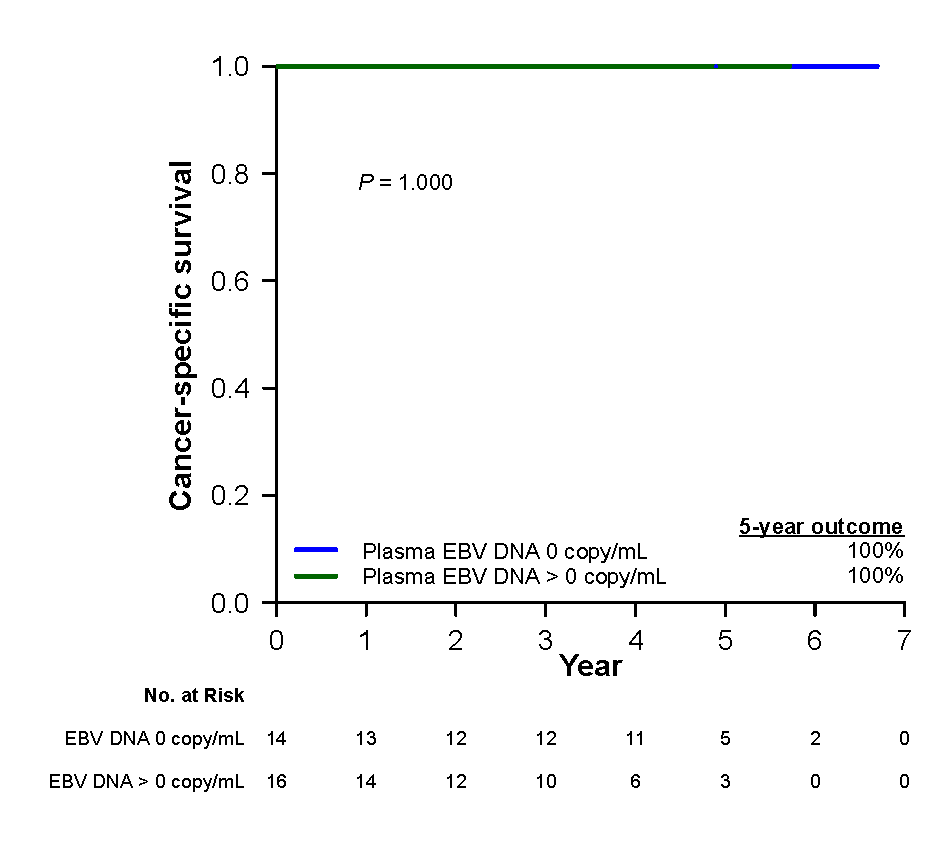
**

**b**

**
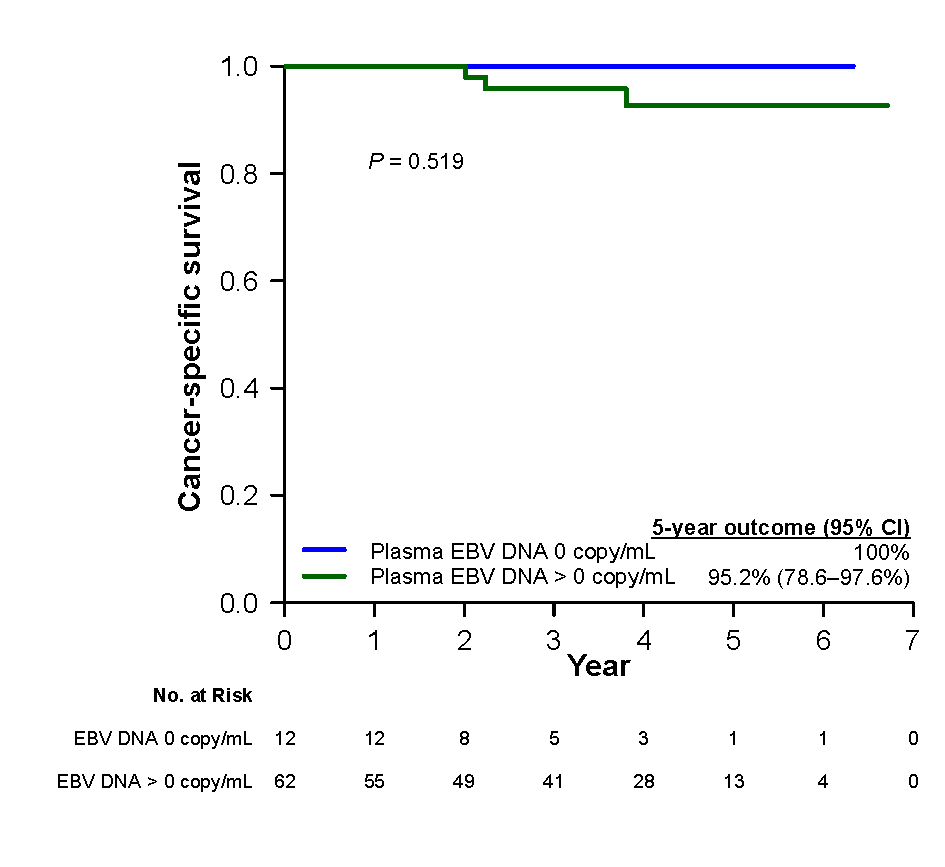
**

**c**

**
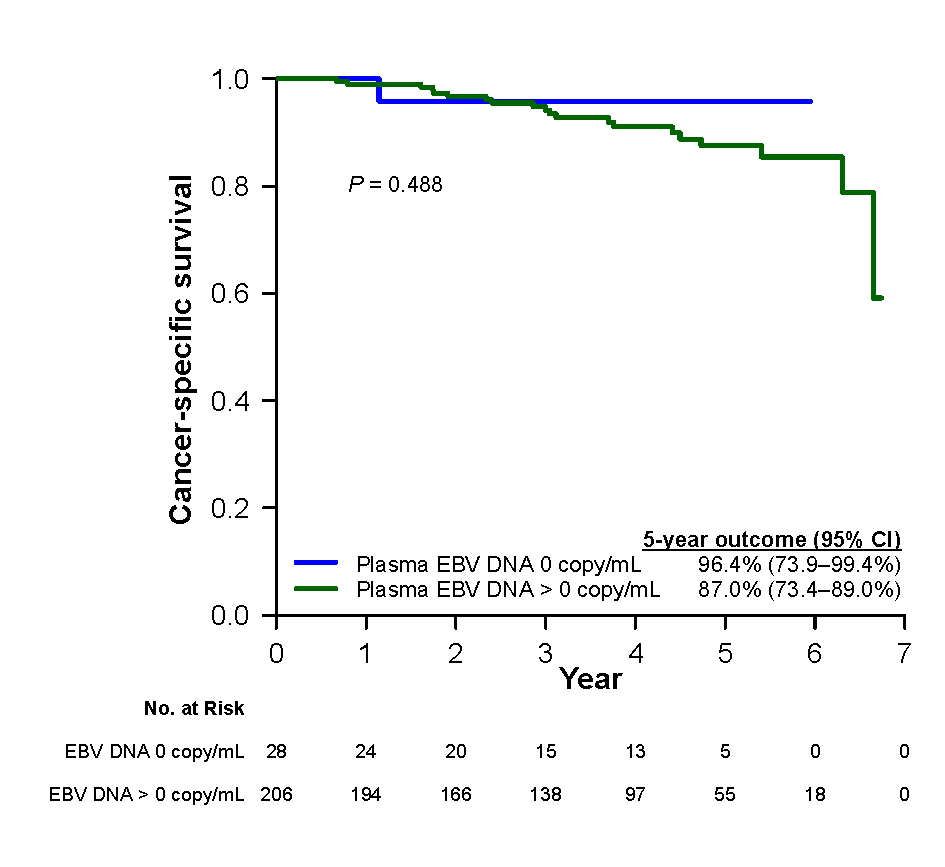
**

**d**

**
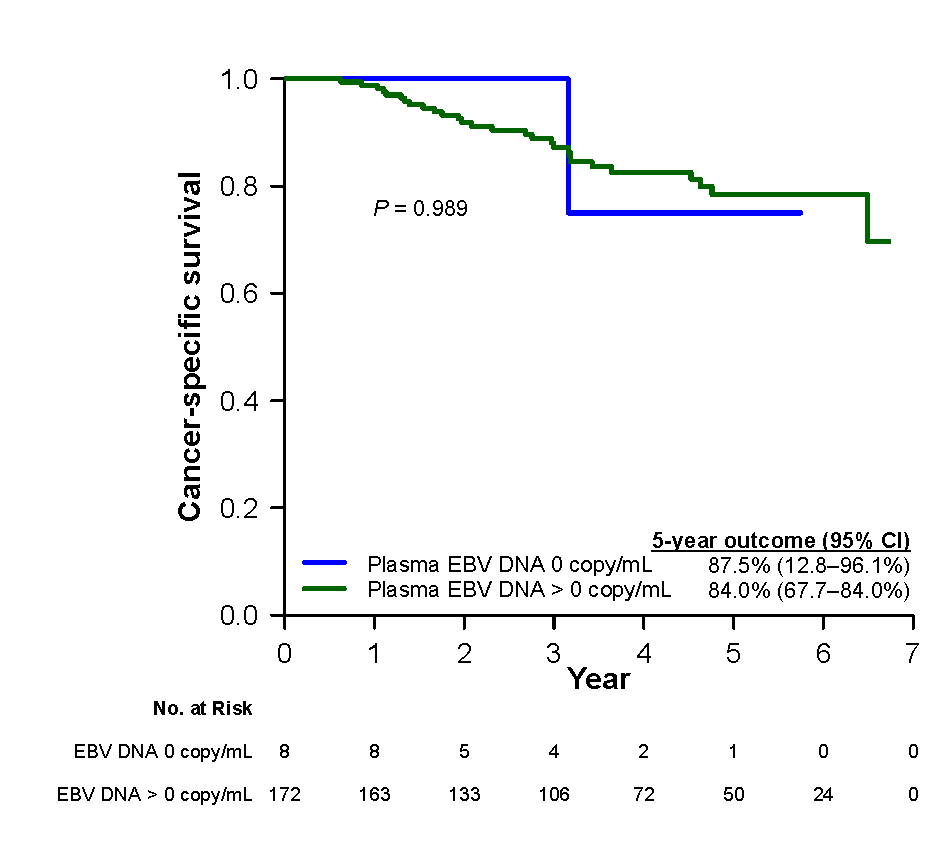
**

**Table S1.** Distribution and T-classification and N-classification of plasma EBV DNA-negative NPC patients based on 8^th^ edition of AJCC/UICC staging classification (*n* = 78)

| **AJCC/UICC 8^th^ edition staging classification** | | | | | |
| --- | --- | --- | --- | --- | --- |
|  | N0 | N1 | N2 | N3 | Total |
| T1 | 18 | 12 | 7 | 3 | 40 |
| T2 | 1 | 4 | 2 | 1 | 8 |
| T3 | 6 | 5 | 14 | 1 | 26 |
| T4 | 0 | 0 | 4 | 0 | 4 |
| Subtotal | 25 | 21 | 27 | 5 | 78 |

*AJCC* American Joint Committee on Cancer, *EBV DNA* Epstein-Barr virus deoxyribonucleic acid, *NPC* nasopharyngeal carcinoma, *UICC* Union for International Cancer Control

**Table S2.** Pre-specified survival endpoints of NPC patients stratified by pre-treatment plasma EBV DNA (0–20 copies/ml vs > 20 copies/ml) and disease stage

| **Pre-treatment plasma EBV DNA (copies/ml)** | **Progression-free Survival** | | **Overall Survival** | | **Cancer-specific Survival** | |
| --- | --- | --- | --- | --- | --- | --- |
|  | 5-year (%) | 95% CI | 5-year (%) | 95% CI | 5-year (%) | 95% CI |
| **Overall study population** | | | | | | |
| 0–20 | 92.7% | 83.3–97.2% | 91.9% | 81.1–97.1% | 96.2% | 85.2–99.0% |
| **>** 20 | 70.0% | 73.1–82.8% | 78.1% | 73.1–82.8% | 84.6% | 80.0–88.3% |
| *p* | 0.023 | | 0.242 | | 0.293 | |
| **Stage I** | | | | | | |
| 0–20 | 94.4% | | 94.4% | | 100.0% | |
| **>** 20 | 75.0% | | 75.0% | | 100.0% | |
| *p* | 0.614 | | 0.614 | | 1.000 | |
| **Stage II** | | | | | | |
| 0–20 | 92.9% | | 90.9% | | 100.0% | |
| **>** 20 | 71.6% | | 81.3% | | 91.9% | |
| *p* | 0.182 | | 0.536 | | 0.368 | |
| **Stage III** | | | | | | |
| 0–20 | 94.0% | | 93.8% | | 96.7% | |
| **>** 20 | 75.2% | | 81.3% | | 87.4% | |
| *p* | 0.143 | | 0.446 | | 0.424 | |
| **Stage IVA** | | | | | | |
| 0–20 | 83.3% | | 80.0% | | 80.0% | |
| **>** 20 | 62.7% | | 73.1% | | 78.2% | |
| *p* | 0.280 | | 0.647 | | 0.815 | |

*CI* confidence interval, *EBV DNA* Epstein-Barr virus deoxyribonucleic acid, *NPC* nasopharyngeal carcinoma

**Table S3.** Distribution and T-classification and N-classification of NPC patients based on 8^th^ edition of AJCC/UICC staging classification who had 0 copy/ml of pre-treatment plasma EBV DNA (*n* = 62)

| **AJCC/UICC 8^th^ edition staging classification** | | | | | |
| --- | --- | --- | --- | --- | --- |
|  | N0 | N1 | N2 | N3 | Total |
| T1 | 14 | 9 | 6 | 3 | 32 |
| T2 | 1 | 2 | 2 | 1 | 6 |
| T3 | 5 | 5 | 10 | 1 | 21 |
| T4 | 0 | 0 | 3 | 0 | 3 |
| Subtotal | 20 | 16 | 21 | 5 | 62 |

*AJCC* American Joint Committee on Cancer, *EBV DNA* Epstein-Barr virus deoxyribonucleic acid, *NPC* nasopharyngeal carcinoma, *UICC* Union for International Cancer Control

**Table S4.** Distribution and T-classification and N-classification of NPC patients based on 8^th^ edition of AJCC/UICC staging classification who had 1-20 copies/ml of pre-treatment plasma EBV DNA (*n* = 16)

| **AJCC/UICC 8^th^ edition staging classification** | | | | | |
| --- | --- | --- | --- | --- | --- |
|  | N0 | N1 | N2 | N3 | Total |
| T1 | 4 | 3 | 1 | 0 | 8 |
| T2 | 0 | 2 | 0 | 0 | 2 |
| T3 | 1 | 0 | 4 | 0 | 5 |
| T4 | 0 | 0 | 1 | 0 | 1 |
| Subtotal | 5 | 5 | 6 | 0 | 16 |

*AJCC* American Joint Committee on Cancer, *EBV DNA* Epstein-Barr virus deoxyribonucleic acid, *NPC* nasopharyngeal carcinoma, *UICC* Union for International Cancer Control

**Table S5.** Patient characteristics at baseline based on 8th edition of AJCC/UICC staging classification stratified by pre-treatment plasma EBV DNA (0 copy/ml vs > 0 copy/ml)

| **Characteristic** | **Patients, No. (%)** | | | ***p*** |
| --- | --- | --- | --- | --- |
|  | **Total (*N* = 518)** | **Pre-treatment plasma EBV DNA** | |  |
|  |  | **0 copy/ml**  **(*n* = 62)** | **> 0 copy/ml**  **(*n* = 456)** |  |
| Median age in years (Range) | 53 (16–90) | 55.5 (16–86) | 53 (16–90) | 0.906 |
| Male/female | 385 (74.3) / 133 (25.7) | 49 (79) /13 (21) | 336 (73.7)/120 (26.3) | 0.366 |
| Histology |  |  |  | 0.500 |
| Keratinising squamous cell carcinoma | 1 (0.2) | 0 (0) | 1 (0.2) |  |
| Non-keratinising differentiated carcinoma | 9 (1.7) | 0 (0) | 9 (2.0) |  |
| Non-keratinising undifferentiated carcinoma | 508 (98.1) | 62 (100) | 446 (97.8) |  |
| ECOG performance status |  |  |  | 0.829 |
| 0 | 80 (15.4) | 9 (14.5) | 71 (15.6) |  |
| 1 | 438 (84.6) | 53 (85.5) | 385 (84.4) |  |
| T-classification |  |  |  | < 0.001 |
| T1 | 147 (28.4) | 32 (51.6) | 115 (25.2) |  |
| T2 | 72 (13.9) | 6 (9.7) | 66 (14.5) |  |
| T3 | 234 (45.2) | 21 (33.9) | 213 (46.7) |  |
| T4 | 65 (12.5) | 3 (4.8) | 62 (13.6) |  |
| N-classification |  |  |  | < 0.001 |
| N0 | 60 (11.6) | 20 (32.3) | 40 (8.8) |  |
| N1 | 127 (24.5) | 16 (25.8) | 111 (24.3) |  |
| N2 | 201 (38.8) | 21 (33.9) | 180 (39.5) |  |
| N3 | 130 (25.1) | 5 (8.1) | 125 (27.4) |  |
| Overall stage |  |  |  | < 0.001 |
| I | 30 (5.8) | 14 (22.6) | 16 (3.5) |  |
| II | 74 (14.3) | 12 (19.4) | 62 (13.6) |  |
| III | 234 (45.2) | 28 (45.2) | 206 (45.2) |  |
| IVA | 180 (34.7) | 8 (12.9) | 172 (37.7) |  |
| Laterality of primary tumour |  |  |  | 0.752 |
| Midline | 231 (44.6) | 28 (45.2) | 203 (44.5) |  |
| Left | 160 (30.9) | 21 (33.9) | 139 (30.5) |  |
| Right | 127 (24.5) | 13 (21) | 114 (25) |  |
| Involvement of retropharyngeal node | 388 (74.9) | 34 (54.8) | 354 (77.6) | < 0.001 |
| Median pre-treatment plasma EBV DNA in copies/millilitre (Range) | 588.5 (0–1143750) | 0 | 848.5 (10–1143750) | 0.214 |
| Stage I | 12 (0–315) | 0 | 42.5 (11–315) | 0.003 |
| Stage II | 321 (0–8850) | 0 | 434 (11–8850) | < 0.001 |
| Stage III | 494  (0–175000) | 0 | 686.5 (10–175000) | < 0.001 |
| Stage IVA | 2012.5 (0–1143750) | 0 | 2164 (14–1143750) | 0.606 |
| Median pre-treatment serum lactate dehydrogenase in international units/litre (range) | 196  (109–688) | 179.5 (125-310) | 197  (109–688) | 0.016 |
| Stage I | 179.5 (121–310) | 178.5 (132–310) | 191 (121–260) | 0.504 |
| Stage II | 185.5 (140–275) | 174.5 (166–250) | 187 (140–275) | 0.259 |
| Stage III | 197.5 (109–521) | 189 (137–306) | 198 (109–521) | 0.442 |
| Stage IVA | 200 (125–688) | 191 (125–254) | 202 (130–688) | 0.239 |
| Median gross tumour volume of the primary tumour (GTV_P) (cm^3^) (Range) | 8.7 (0–191.3) | 9.4 (0.4–191.3) | 18.2 (0–168.2) | 0.020 |
| Median gross tumour volume of the positive neck nodes (GTV_N) (cm^3^) (Range) | 17.1 (0–136) | 4.85 (0–66.9) | 9.8 (0–136) | < 0.001 |
| Median gross tumour volume of the primary tumour and the positive neck nodes (GTV_P+N) (cm^3^) (Range) | 31.4  (0.9–229) | 16.35  (2.3–199.9) | 33.8  (0.9–229) | < 0.001 |
| Radical IMRT only | 71 (13.7) | 22 (35.5) | 49 (10.7) | < 0.001 |
| Concurrent chemoradiation | 91 (17.6) | 10 (16.1) | 81 (17.8) | 0.421 |
| Induction chemotherapy then concurrent chemoradiation | 165 (31.9) | 10 (16.1) | 155 (34) | 0.085 |
| Concurrent chemoradiation then adjuvant chemotherapy | 191 (36.9) | 20 (32.3) | 171 (37.5) | 0.394 |

*AJCC* American Joint Committee on Cancer, *EBV DNA* Epstein-Barr virus deoxyribonucleic acid, *ECOG* Eastern Cooperative Oncology Group, *IMRT* intensity-modulated radiation therapy, *UICC* Union for International Cancer Control

**Table S6.** Patient characteristics at baseline based on 8th edition of AJCC/UICC staging classification stratified by pre-treatment plasma EBV DNA (0 copy/ml vs 1-20 copies/ml vs > 20 copies/ml)

| **Characteristic** | **Patients, No. (%)** | | | | ***p*** |
| --- | --- | --- | --- | --- | --- |
|  | **Total  (*N* = 518)** | **Pre-treatment plasma EBV DNA** | | |  |
|  |  | **0 copy/ml**  **(*n* = 62)** | **1-20 copies/ml**  **(*n* = 16)** | **> 20 copies/ml**  **(*n* = 440)** |  |
| Median age in years (Range) | 53  (16–90) | 55.5  (16–86) | 50  (35–78) | 53 (16-90) | 0.850 |
| Male/female | 385 (74.3) / 133 (25.7) | 49 (79) /13 (21) | 12 (75.0)/4 (25.0) | 324 (73.6)/ 116 (26.4) | 0.659 |
| Histology |  |  |  |  | 0.518 |
| Keratinising squamous cell carcinoma | 1 (0.2) | 0 (0) | 0 (0) | 1 (0.2) |  |
| Non-keratinising differentiated carcinoma | 9 (1.7) | 0 (0) | 0 (0) | 9 (2.0) |  |
| Non-keratinising undifferentiated carcinoma | 508 (98.1) | 62 (100) | 16 (100) | 430 (97.7) |  |
| ECOG performance status |  |  |  |  | 0.205 |
| 0 | 80 (15.4) | 9 (14.5) | 5 (31.3) | 66 (15.0) |  |
| 1 | 438 (84.6) | 53 (85.5) | 11 (68.8) | 374 (85.0) |  |
| T-classification |  |  |  |  | < 0.001 |
| T1 | 147 (28.4) | 32 (51.6) | 8 (50.0) | 107 (24.3) |  |
| T2 | 72 (13.9) | 6 (9.7) | 2 (12.5) | 64 (14.5) |  |
| T3 | 234 (45.2) | 21 (33.9) | 5 (31.3) | 208 (47.3) |  |
| T4 | 65 (12.5) | 3 (4.8) | 1 (6.3) | 61 (13.9) |  |
| N-classification |  |  |  |  | < 0.001 |
| N0 | 60 (11.6) | 20 (32.3) | 5 (31.3) | 35 (8.0) |  |
| N1 | 127 (24.5) | 16 (25.8) | 5 (31.3) | 106 (24.1) |  |
| N2 | 201 (38.8) | 21 (33.9) | 6 (37.5) | 174 (39.5) |  |
| N3 | 130 (25.1) | 5 (8.1) | 0 (0) | 125 (28.4) |  |
| Overall stage |  |  |  |  | < 0.001 |
| I | 30 (5.8) | 14 (22.6) | 4 (25.0) | 12 (2.7) |  |
| II | 74 (14.3) | 12 (19.4) | 5 (131.3) | 57 (13.0) |  |
| III | 234 (45.2) | 28 (45.2) | 6 (37.5) | 200 (45.5) |  |
| IVA | 180 (34.7) | 8 (12.9) | 1 (6.3) | 171 (38.9) |  |
| Laterality of primary tumour |  |  |  |  | 0.966 |
| Midline | 231 (44.6) | 28 (45.2) | 7 (43.8) | 196 (44.5) |  |
| Left | 160 (30.9) | 21 (33.9) | 5 (31.3) | 134 (30.5) |  |
| Right | 127 (24.5) | 13 (21) | 4 (25.0) | 110 (25.0) |  |
| Involvement of retropharyngeal node | 388 (74.9) | 34 (54.8) | 9 (56.3) | 345 (78.4) | < 0.001 |
| Median pre-treatment plasma EBV DNA in copies/millilitre (Range) | 588.5  (0–1143750) | 0 | 13.5  (10–19) | 895 (22–1143750) | < 0.001 |
| Stage I | 12 (0–315) | 0 | 15.5 (11–19) | 64 (24–315) | < 0.001 |
| Stage II | 321 (0–8850) | 0 | 11  (11–16) | 547  (22–8850) | < 0.001 |
| Stage III | 494  (0–175000) | 0 | 15  (10–17) | 705.5 (22–175000) | < 0.001 |
| Stage IVA | 2012.5  (0–1143750) | 0 | 14 | 2203 (38–1143750) | < 0.001 |
| Median pre-treatment serum lactate dehydrogenase in international units/litre (range) | 196  (109–688) | 179.5 (125-310) | 184  (143–276) | 198 (109–688) | 0.008 |
| Stage I | 179.5 (121–310) | 178.5 (132–310) | 202.5 (151–230) | 190.5 (121–260) | 0.636 |
| Stage II | 185.5  (140–275) | 174.5 (166–250) | 172  (143–275) | 188 (140–256) | 0.247 |
| Stage III | 197.5  (109–521) | 189  (137–306) | 199  (149–276) | 198 (109–521) | 0.584 |
| Stage IVA | 200  (125–688) | 191  (125–254) | 165 | 204 (130–688) | 0.242 |
| Median gross tumour volume of the primary tumour (GTV_P) (cm^3^) (Range) | 8.7  (0–191.3) | 9.4  (0.4–191.3) | 9.85  (2.4–37.9) | 10.1 (0–136) | < 0.001 |
| Median gross tumour volume of the positive neck nodes (GTV_N) (cm^3^) (Range) | 17.1  (0–136) | 4.85  (0–66.9) | 2.55  (0–22.4) | 18.55 (0–136) | < 0.001 |
| Median gross tumour volume of the primary tumour and the positive neck nodes (GTV_P+N) (cm^3^) (Range) | 31.4  (0.9–229) | 16.35  (2.3–199.9) | 16.05  (3.1–49.5) | 34.9  (0.9–229) | < 0.001 |
| Radical IMRT only | 71 (13.7) | 22 (35.5) | 4 (25.0) | 45 (10.2) | < 0.001 |
| Concurrent chemoradiation | 91 (17.6) | 10 (16.1) | 4 (33.3) | 77 (19.2) | 0.353 |
| Induction chemotherapy then concurrent chemoradiation | 165 (31.9) | 10 (16.1) | 2 (16.7) | 153 (34.7) | 0.062 |
| Concurrent chemoradiation then adjuvant chemotherapy | 191 (36.9) | 20 (32.3) | 7 (58.3) | 164 (37.2) | 0.394 |

*AJCC* American Joint Committee on Cancer, *EBV DNA* Epstein-Barr virus deoxyribonucleic acid, *ECOG* Eastern Cooperative Oncology Group, *IMRT* intensity-modulated radiation therapy, *UICC* Union for International Cancer Control

**Table S7.** Pre-specified survival endpoints of NPC patients stratified by pre-treatment plasma EBV DNA (0 copy/ml vs > 0 copy/ml) and disease stage

| **Pre-treatment plasma EBV DNA (copies/ml)** | **Progression-free survival** | | **Overall survival** | | **Cancer-specific survival** | |
| --- | --- | --- | --- | --- | --- | --- |
|  | 5-year (%) | 95% CI | 5-year (%) | 95% CI | 5-year (%) | 95% CI |
| **Overall study population** | | | | | | |
| 0 | 90.8% | 79.0–96.0% | 89.9% | 77.0–96.0% | 95.4% | 82.0–99.0% |
| > 0 | 70.8% | 65.0–76.0% | 78.6% | 73.0–83.0% | 85.0% | 80.0–99.0% |
| *p* | 0.016 | | 0.196 | | 0.092 | |
| **Stage I** | | | | | | |
| 0 | 92.9% | | 92.9% | | 100.0% | |
| > 0 | 80.0% | | 80.0% | | 100.0% | |
| *p* | 0.846 | | 0.846 | | 1.000 | |
| **Stage II** | | | | | | |
| 0 | 88.9% | | 83.3% | | 100.0% | |
| > 0 | 74.0% | | 82.8% | | 92.7% | |
| *p* | 0.474 | | 0.966 | | 0.519 | |
| **Stage III** | | | | | | |
| 0 | 92.7% | | 92.4% | | 95.8% | |
| > 0 | 75.5% | | 81.4% | | 87.5% | |
| *p* | 0.221 | | 0.546 | | 0.488 | |
| **Stage IVA** | | | | | | |
| 0 | 80.0% | | 75.0% | | 75.0% | |
| > 0 | 63.0% | | 73.4% | | 78.4% | |
| *p* | 0.390 | | 0.819 | | 0.989 | |

*CI* confidence interval; *EBV DNA* Epstein-Barr virus deoxyribonucleic acid, *NPC* nasopharyngeal carcinoma

**Table S8.** Cross-tabulation of EBER scores and T-stage, N-stage and overall stage of nasopharyngeal carcinoma in the whole study population (n= 518)

|  | | T-Stage | | | | *p* | N-Stage | | | | *p* | Overall Stage | | | | *p* | Total |
| --- | --- | --- | --- | --- | --- | --- | --- | --- | --- | --- | --- | --- | --- | --- | --- | --- | --- |
|  |  | 1 | 2 | 3 | 4 |  | 0 | 1 | 2 | 3 |  | I | II | III | IVA |  |  |
| EBER Score | 0–1 | 5 | 2 | 9 | 4 | 0.568 | 0 | 3 | 7 | 10 | 0.056 | 0 | 1 | 6 | 13 | 0.004 | 20 |
|  | 2 | 16 | 9 | 16 | 4 |  | 8 | 12 | 19 | 6 |  | 7 | 8 | 20 | 10 |  | 45 |
|  | 3 | 126 | 61 | 209 | 57 |  | 52 | 112 | 175 | 114 |  | 23 | 65 | 208 | 157 |  | 453 |
| Total | | 147 | 72 | 234 | 65 |  | 60 | 127 | 201 | 130 |  | 30 | 74 | 234 | 180 |  | 518 |

*EBER* Epstein-Barr virus encoded RNA

**Table S9.** Cross-tabulation of EBER scores and T-stage, N-stage and overall stage of nasopharyngeal carcinoma in patients with pre-treatment plasma EBV DNA 0–20 copies/ml (*n* = 78)

|  | | T-Stage | | | | *p* | N-Stage | | | | *p* | Overall Stage | | | | *p* | Total |
| --- | --- | --- | --- | --- | --- | --- | --- | --- | --- | --- | --- | --- | --- | --- | --- | --- | --- |
|  |  | 1 | 2 | 3 | 4 |  | 0 | 1 | 2 | 3 |  | I | II | III | IVA |  |  |
| EBER Score | 0–1 | 2 | 1 | 2 | 1 | 0.479 | 0 | 2 | 3 | 1 | 0.182 | 0 | 1 | 3 | 2 | 0.518 | 6 |
|  | 2 | 11 | 4 | 8 | 2 |  | 5 | 7 | 12 | 1 |  | 5 | 5 | 12 | 3 |  | 25 |
|  | 3 | 27 | 3 | 16 | 1 |  | 20 | 12 | 12 | 3 |  | 13 | 11 | 19 | 4 |  | 47 |
| Total | | 40 | 8 | 26 | 4 |  | 25 | 21 | 27 | 5 |  | 18 | 17 | 34 | 9 |  | 78 |

*EBER* Epstein-Barr virus encoded RNA, *EBV DNA* Epstein-Barr virus deoxyribonucleic acid

**Table S10.** Cross-tabulation of EBER scores and T-stage, N-stage and overall stage of nasopharyngeal carcinoma in patients with pre-treatment plasma EBV DNA > 20 copies/ml (*n* = 440)

|  | | T-Stage | | | | *p* | N-Stage | | | | *p* | Overall Stage | | | | *p* | Total |
| --- | --- | --- | --- | --- | --- | --- | --- | --- | --- | --- | --- | --- | --- | --- | --- | --- | --- |
|  |  | 1 | 2 | 3 | 4 |  | 0 | 1 | 2 | 3 |  | I | II | III | IVA |  |  |
| EBER Score | 0–1 | 3 | 1 | 7 | 3 | 0.790 | 0 | 1 | 4 | 9 | 0.081 | 0 | 0 | 3 | 11 | 0.027 | 14 |
|  | 2 | 5 | 5 | 8 | 2 |  | 3 | 5 | 7 | 5 |  | 2 | 3 | 8 | 7 |  | 20 |
|  | 3 | 99 | 58 | 193 | 56 |  | 32 | 100 | 163 | 111 |  | 10 | 54 | 189 | 153 |  | 406 |
| Total | | 107 | 64 | 208 | 61 |  | 34 | 106 | 174 | 125 |  | 12 | 57 | 200 | 171 |  | 440 |

*EBER* Epstein-Barr virus encoded RNA, *EBV DNA* Epstein-Barr virus deoxyribonucleic acid

**Table S11.** Cross-tabulation of EBER scores and pre-treatment plasma EBV DNA (0–20 copies/ml vs > 20 copies/ml, and 0 copy/ml vs > 0 copy/ml)

|  | | EBER Score | | | Total | *p* |
| --- | --- | --- | --- | --- | --- | --- |
|  |  | 0–1 | 2 | 3 |  |  |
| EBV DNA (copies/ml) | 0–20 | 6 | 25 | 47 | 78 | < 0.001 |
|  | > 20 | 14 | 20 | 406 | 440 |  |
|  | 0 | 6 | 15 | 41 | 62 | < 0.001 |
|  | > 0 | 14 | 30 | 412 | 456 |  |
| Total | | 20 | 45 | 453 | 518 |  |

*EBER* Epstein-Barr virus encoded RNA, *EBV DNA* Epstein-Barr virus deoxyribonucleic acid

**Table S12.** Cross-tabulation of EBER scores and T-stage, N-stage and overall stage of nasopharyngeal carcinoma in patients with pre-treatment plasma EBV DNA 0 copy/ml (*n* = 62)

|  | | T-Stage | | | | *p* | N-Stage | | | | *p* | Overall Stage | | | | *p* | Total |
| --- | --- | --- | --- | --- | --- | --- | --- | --- | --- | --- | --- | --- | --- | --- | --- | --- | --- |
|  |  | 1 | 2 | 3 | 4 |  | 0 | 1 | 2 | 3 |  | I | II | III | IVA |  |  |
| EBER Score | 0–1 | 2 | 1 | 2 | 1 | 0.615 | 0 | 2 | 3 | 1 | 0.156 | 0 | 1 | 3 | 2 | 0.365 | 6 |
|  | 2 | 6 | 2 | 6 | 1 |  | 2 | 4 | 8 | 1 |  | 2 | 2 | 9 | 2 |  | 15 |
|  | 3 | 24 | 3 | 13 | 1 |  | 18 | 10 | 10 | 3 |  | 12 | 9 | 16 | 4 |  | 41 |
| Total | | 32 | 6 | 21 | 3 |  | 20 | 16 | 21 | 5 |  | 14 | 12 | 28 | 8 |  | 62 |

*EBER* Epstein-Barr virus encoded RNA, *EBV DNA* Epstein-Barr virus deoxyribonucleic acid

**Table S13.** Cross-tabulation of EBER scores and T-stage, N-stage and overall stage of nasopharyngeal carcinoma in patients with pre-treatment plasma EBV DNA > 0 copy/ml (*n* = 456)

|  | | T-Stage | | | | *p* | N-Stage | | | | *p* | Overall Stage | | | | *p* | Total |
| --- | --- | --- | --- | --- | --- | --- | --- | --- | --- | --- | --- | --- | --- | --- | --- | --- | --- |
|  |  | 1 | 2 | 3 | 4 |  | 0 | 1 | 2 | 3 |  | I | II | III | IVA |  |  |
| EBER Score | 0–1 | 3 | 1 | 7 | 3 | 0.512 | 0 | 1 | 4 | 9 | 0.013 | 0 | 0 | 3 | 11 | < 0.001 | 14 |
|  | 2 | 10 | 7 | 10 | 3 |  | 6 | 8 | 11 | 5 |  | 5 | 6 | 11 | 8 |  | 30 |
|  | 3 | 102 | 58 | 196 | 56 |  | 34 | 102 | 165 | 111 |  | 11 | 56 | 192 | 153 |  | 412 |
| Total | | 115 | 66 | 213 | 62 |  | 40 | 111 | 180 | 125 |  | 16 | 62 | 206 | 172 |  | 456 |

*EBER* Epstein-Barr virus encoded RNA, *EBV DNA* Epstein-Barr virus deoxyribonucleic acid
